# Equivalent analytical model for liquid sloshing in a 2-D rectangular container with multiple vertical baffles by subdomain partition approach

**DOI:** 10.1038/s41598-024-63781-7

**Published:** 2024-06-05

**Authors:** Ying Sun, Xun Meng, Zhong Zhang, Zhenyuan Gu, Jiadong Wang, Ding Zhou

**Affiliations:** 1 School of Transportation and Civil Engineering, Nantong University, Nantong, China; 2 College of Civil Engineering, Yancheng Institute of Technology, Yancheng, China; 3 Faculty of Civil Engineering and Mechanics, Jiangsu University, Zhenjiang, China; 4 College of Civil Engineering, Nanjing Tech University, Nanjing, China

**Keywords:** Rectangular container, Vertical baffles, Analytical model, Liquid sloshing, Dynamic response, Subdomain partition approach, Civil engineering, Fluid dynamics, Applied mathematics

## Abstract

An equivalent analytical model of sloshing in a two-dimensional (2-D) rigid rectangular container equipped with multiple vertical baffles is presented. Firstly, according to the subdomain partition approach, the total liquid domain is partitioned into subdomains with the pure interface and boundary conditions. The separation of variables is utilized to achieve the velocity potential for subdomains. Then, sloshing characteristics are solved according to continuity and free surface conditions. According to the mode orthogonality of sloshing, the governing motion equation for sloshing under horizontal excitation is given by introducing generalized time coordinates. Besides, by producing the same hydrodynamic shear and overturning moment as those from the original container-liquid-baffle system, a mass-spring analytical model of the continuous liquid sloshing is established. The equivalent masses and corresponding locations are presented in the model. The feasibility of the present approach is verified by conducting comparative investigations. Finally, by utilizing normalized equivalent model parameters, the sloshing behaviors of the baffled container are investigated regarding baffle positions and heights as well as the liquid height, respectively.

## Introduction

The rectangular liquid storage container is of great significance due to its extensive applications in energy storage and transportation, ocean engineering, water supply and sewage treatment as well as nuclear power plant. Serious structural failure for liquid storage containers may occur due to the additional forces acting on the container wall caused by liquid oscillation^1–3^. Therefore, it is of great benefit to study liquid-structure interaction of systems, which can help improve the system safety under external seismic loads and reduce the probability of structure failure^4–7^. Luo et al.^8^ carried out the studies of stratified sloshing in a partially-filled rectangular container. Park et al.^9^ used an experiment technique to obtain liquid vibration mode and dynamic performance of a storage container. Considering shell-liquid and shell-wind interactions, Jing et al.^10^ proposed a refined calculation model to investigate responses in tanks undergoing the seismic excitation and wind load. Balasubramanian et al.^11^ analyzed forced vibrations of a liquid-filled circular shell experimentally. Tsao et al.^12,13^ obtained dynamic properties and sloshing damping of liquid in rectangular and cylindrical containers occupied by porous media.

Scholars focused on the higher improvement in the vibration reduction behavior under harmonic and seismic excitations^14–16^; for instance, in order to mitigate the structure vibration caused by the sloshing oscillation under seismic excitation, anti-sloshing baffles with various configurations are installed inside the storage containers^17^. Numerous investigations using numerical and experimental methods can be found on evaluating efficiency of internal baffles in reducing sloshing in containers. Xue and Lin^18^ numerically obtained baffle impacts on diminishing sloshing. Huang^19^ performed a comprehensive numerical study on fluid dynamics in a baffled storage container. By using the boundary element approach, the natural frequency and vibration mode in arbitrary shaped liquid storage systems with rigid baffles were studied^20^. Sanapala et al.^21^ numerically simulated large amplitude motion in a 2-D liquid storage container with bilateral baffles undergoing vertical excitation as well as seismic excitation. The experimental researches can also reveal complicated variation laws and mechanical phenomena of liquid sloshing. Xue et al.^22^ experimentally investigated baffle effects on sloshing mitigation in a rectangular container. Ren et al.^23^ studied sloshing properties in a baffled container experimentally. Cho and Kim^24^ experimentally and theoretically analyzed influences of baffles on sloshing motions in a rectangular container. Yu et al.^25^ experimentally determined coupling vibrations of baffles and fluid in a shallow container undergoing horizontal excitations. The above-mentioned works were mainly about numerical or experimental investigations. However, the numerical solutions may be influenced by the meshing accuracy; and the experimental methods could be limited by the high cost under seismic excitation.

A parametric study can be easily to be conducted to evaluate baffle influences on suppressing the sloshing in terms of analytical or semi-analytical methods^26^. Goudarzi and Sabbagh-Yazdi^27^ made the assessment on effectivity of baffles in storage containers analytically. Cheng et al.^28^ acquired the sloshing response in a storage structure with baffle using a simplified calculation approach. Meng et al.^29^ obtained baffle impacts on vibrations of sloshing in a rectangular container. Cho^30^ developed an analytical model to determine mitigation impacts of the baffle in a rectangular container. Wang et al.^31^ evaluated the sloshing mitigation in a cylindrical container with baffle by an analytical subdomain partition approach. According to the subdomain partition approach, Sun et al.^32^ established an analytical mechanical model with mass-springs to replace the continuous liquid in the baffled cylindrical containers.

As described above, the existing numerical and analytical researches on dynamics of baffled rectangular containers could require intensive calculation, especially for complicated liquid storage systems. In the present paper, an analytical model of a horizontally excited 2-D rectangular container with multiple rigid vertical baffles is proposed to simplify dynamic investigations of the system with accurate solutions and small computational cost, which is the novelty of the present work. The velocity potential of liquid is acquired based on the subdomain partition method. Through producing the same hydrodynamic shear and moment as those of the original container-liquid-baffle system, a dynamic analytical model is constructed to substitute for the sloshing of continuous liquid. Detailed parametric study of structural dynamics is conducted with respond to the baffle location and height as well as liquid height.

## Mathematical background

**Figure 1:**
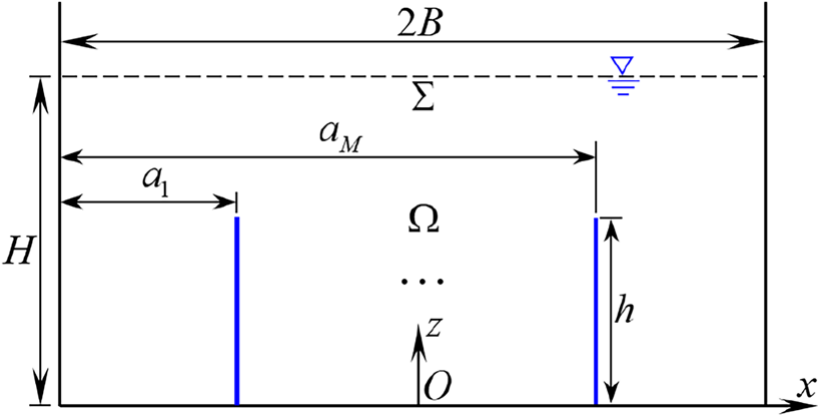
A vertically-baffled 2-D rectangular storage container partially full of liquid.

**Figure 2:**
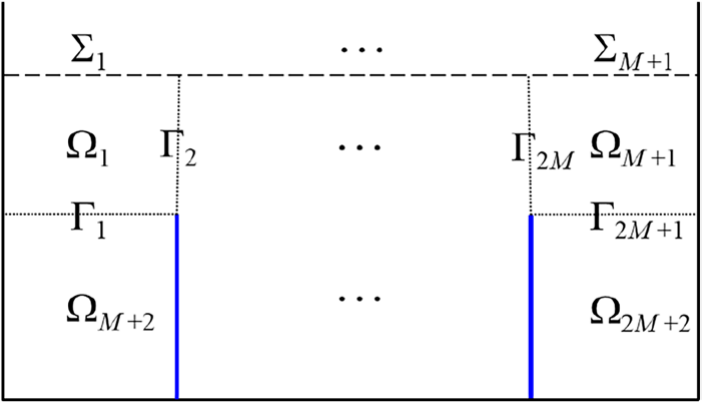
Liquid subdomain partition and corresponding artificial interfaces.

Figure 1 depicts a rigid 2-D rectangular container with the multiple rigid vertical baffles. The origin of the coordinate system *Oxz* is positioned at bottom center of the container. The storage container is partly full of incompressible, irrotational and inviscid liquid with depth *H* and width 2*B*. The linear equation is utilized under the circumstance of the small sloshing amplitude compared with the container cross-section size. The thicknesses of the container and baffles are negligible. The multiple baffles are rigidly mounted at container bottom with the baffle height *h*. The distance from the left container wall to *M*th baffle is defined as $a_{M} .$ $a_{0}$ represents the left wall position. $a_{M + 1}$ denotes the position of the right wall of the container. The total liquid domain Ω is divided into several subdomains $\Omega_{i} \;(i = 1,\;2, \ldots ,\;2M + 2)$ with the $2M + 1$ artificial interfaces on the basis of the subdomain partition approach presented by Wang et al.^31^ in Fig. 2. The *M* + 1 horizontal artificial interfaces are, respectively, defined as $\Gamma_{1}$, $\Gamma_{3}$, …, $\Gamma_{2M + 1}$; the *M* vertical artificial interfaces are, respectively, defined as $\Gamma_{2}$, $\Gamma_{4}$, …, $\Gamma_{2M} .$ $\Sigma_{i}$ is the free surface of the subdomains $\Omega_{i} \;(i = 1,\;2, \ldots ,\;M + 1).$

According to above definitions, the liquid velocity potential function could be expressed as

1 $$ \varphi (x,\;z,\;t) = \varphi_{i} (x,\;z,\;t),\;(x,z) \in \Omega_{i} ,\;(i = 1,2,\;...,2M + 2) $$

in which, $\varphi_{i} (x,\;z,\;t)$ denotes the velocity potential corresponding to $\Omega_{i}$ and should satisfy Laplace equation:

2 $$ \nabla^{2} \varphi_{i} = 0,\;(x,z) \in \Omega_{i} ,\;(i = 1,2,\;...,2M + 2) $$

Considering impermeability conditions at container surfaces, the normal velocity of liquid satisfies the rigid boundary condition:

3 $$ \left. {\frac{{\partial \varphi_{i} }}{\partial z}} \right|_{z = 0} = 0,\;\left. {\frac{{\partial \varphi_{1} }}{\partial x}} \right|_{{x = a_{0} - B}} = 0,\;\left. {\frac{{\partial \varphi_{M + 1} }}{\partial x}} \right|_{{x = a_{M + 1} - B}} = 0 $$

4 $$ \left. {\frac{{\partial \varphi_{i} }}{\partial x}} \right|_{{x = a_{s} - B}} = 0,\;\left. {\frac{{\partial \varphi_{i} }}{\partial x}} \right|_{{x = a_{s + 1} - B}} = 0,\;(i = s + M + 2,s = 0,1,\;...,M) $$

The motion equation of the free surface has

5 $$ \left. {\frac{{\partial \varphi_{i} }}{\partial z}} \right|_{z = H} + \frac{1}{g}\left. {\frac{{\partial^{2} \varphi_{i} }}{{\partial t^{2} }}} \right|_{z = H} = 0,\;(i = 1,2,\;...,2M + 1) $$

where *g* represents the gravity acceleration. To simply represent the relationship between adjacent subdomains $\Omega_{i}$ and $\Omega_{{i^{\prime}}}$ as well as the artificial interface Γ_*k*_, the ordered triple (*i*, *i*′, *k*) meets

6 $$ (i,\;i{\prime} ,\;k) \in \left\{ {\begin{array}{*{20}c} {\{ \left. {(s + 1,\;s + M + 2,\;2s + 1)} \right|(s = 0,\;1,\; \ldots ,\;M)\} } \\ {\{ \left. {(s + 1,\;s + 2,\;2s + 2)} \right|(s = 0,\;1,\; \ldots ,\;M - 1)\} } \\ \end{array} } \right. $$

$\Omega_{i}$ and $\Omega_{{i^{\prime}}}$ should satisfy the continuity condition for pressure and velocity:

7 $$ \frac{{\partial \varphi_{i} }}{\partial t} = \frac{{\partial \varphi_{{i^{\prime}}} }}{\partial t},\;\frac{{\partial \varphi_{i} }}{{\partial {\mathbf{n}}_{k} }} = \frac{{\partial \varphi_{{i^{\prime}}} }}{{\partial {\mathbf{n}}_{k} }},\;{\text{on}}\;\Gamma_{k} $$

in which ${\mathbf{n}}_{k}$ represents normal vector to the interface $\Gamma_{k}$.

## Free vibration of liquid

### Solution to liquid velocity potential

The liquid velocity potential $\varphi_{i} (x,\;z,\;t)$ could be written as $\varphi_{i} (x,\;z,\;t) = {\rm j}\omega {\rm e}^{{{\rm j}\omega t}} \Phi_{i} \left( {x,z} \right),\;(x,z) \in \Omega_{i} ,\;(i = 1,2,\;...,2M + 2)$ on the basis of linearized sloshing theory; $\Phi_{i} (x,z)$ is the vibration mode of subdomain $\Omega_{i}$. Thus, Eqs. (1)–(4) yield

8 $$ \frac{{\partial^{2} \Phi_{i} }}{{\partial x^{2} }} + \frac{{\partial^{2} \Phi_{i} }}{{\partial z^{2} }} = 0,\;(x,z) \in \Omega_{i} ,\;(i = 1,2,\;...,2M + 2) $$

9 $$ \left. {\frac{{\partial \Phi_{i} }}{\partial z}} \right|_{z = 0} = 0,\;\left. {\frac{{\partial \Phi_{1} }}{\partial x}} \right|_{{x = a_{0} - B}} = 0,\;\left. {\frac{{\partial \Phi_{M + 1} }}{\partial x}} \right|_{{x = a_{M + 1} - B}} = 0 $$

10 $$ \left. {\frac{{\partial \Phi_{i} }}{\partial x}} \right|_{{x = a_{s} - B}} = 0,\;\left. {\frac{{\partial \Phi_{i} }}{\partial x}} \right|_{{x = a_{s + 1} - B}} = 0,\;(i = s + M + 2,\;s = 0,1,\;...,M) $$

Based on Eqs. (8)–(10), the mode shape $\Phi_{i}$ can be written as the following form using the superposition principle of potential functions:

11 $$ \Phi_{i} = \sum\limits_{l}^{{K_{i} }} {\Phi_{i}^{l} } ,\;K_{i} = \left\{ {\begin{array}{*{20}l} {1,\;(i = s + M + 2,s = \, 0,1, \, \ldots ,M)} \hfill \\ {3,\;\left( {i = 1,M + 1} \right)} \hfill \\ {4,\;\left( {i = s + 1,s = 1, \ldots ,M{-}1} \right)} \hfill \\ \end{array} } \right. $$

where $K_{i}$ denotes the number of boundary conditions for the subdomain $\Omega_{i}$. $\Phi_{i}^{l}$ is the *l*th kind of the liquid velocity potential. Substituting Eq. (11) into Eqs. (8)–(10) obtains the movement equation and impermeability conditions for the boundary:

12 $$ \frac{{\partial^{2} \Phi_{i}^{l} }}{{\partial x^{2} }} + \frac{{\partial^{2} \Phi_{i}^{l} }}{{\partial z^{2} }} = 0,\;(x,z) \in \Omega_{i} ,\;(i = 1,2,\;...,2M + 2) $$

13 $$ \left. {\frac{{\partial \Phi_{i}^{l} }}{\partial x}} \right|_{{x = a_{s} - B}} = 0,\;\left. {\frac{{\partial \Phi_{i}^{l} }}{\partial x}} \right|_{{x = a_{s + 1} - B}} = 0,\;\left. {\frac{{\partial \Phi_{i}^{l} }}{\partial z}} \right|_{{z = z_{0} }} = 0 $$

14 $$ \left. {\frac{{\partial \Phi_{{i^{\prime}}}^{l} }}{\partial x}} \right|_{{x = a_{s} - B}} = 0,\;\left. {\frac{{\partial \Phi_{{i^{\prime}}}^{l} }}{\partial x}} \right|_{{x = a_{s + 1} - B}} = 0,\;\left. {\Phi_{{i^{\prime}}}^{l} } \right|_{z = H} = 0 $$

in which,$\left( {i,\;z_{0} ,\;l} \right) \in \{ \left( {s + 1,\;h,\;2} \right),\;\left. {\left( {s + M + 2,\;0,\;1} \right)} \right|\left( {s = 0,\;1, \ldots ,M} \right)\} ,$ $(i{\prime} ,\;s,\;l) \in \{ \left. {\left( {s + 1,\;s,\;3} \right)} \right|\left( {s = 0,\;M} \right),\;\left. {\left( {s + 1,\;s, \, 4} \right)} \right|\left( {s = 1,\;2, \ldots ,M - 1} \right)\}$. $\Phi_{i}^{l}$ meets rigid boundary conditions of lower, left and right surfaces. $\Phi_{{i^{\prime}}}^{l}$ meets rigid boundary conditions of left and right surfaces as well as zero-pressure condition on the upper surface.

15 $$ \left. {\frac{{\partial \Phi_{i}^{1} }}{\partial x}} \right|_{{x = a_{s} - B}} = 0,\;\left. {\frac{{\partial \Phi_{i}^{1} }}{\partial z}} \right|_{z = h} = 0,\;\left. {\Phi_{i}^{1} } \right|_{z = H} = 0,\;(i = s + 1,s = 0,1,\;...,M - 1) $$

16 $$ \left. {\frac{{\partial \Phi_{i}^{l} }}{\partial x}} \right|_{{x = a_{s + 1} - B}} = 0,\;\left. {\frac{{\partial \Phi_{i}^{l} }}{\partial z}} \right|_{z = h} = 0,\;\left. {\Phi_{i}^{l} } \right|_{z = H} = 0,\;(i,s,l) \in \{ (s + 1,s,2),(M + 1,M,1)\} $$

In Eq. (16), $s = 1,\;2, \ldots ,M - 1.$ $\Phi_{i}^{1}$ meets rigid boundary conditions of left and lower surfaces as well as zero-pressure boundary condition on the upper surface. $\Phi_{i}^{l}$ meets rigid boundary conditions on right and lower surfaces as well as zero-pressure condition on the upper surface.

To obtain simplification in present analysis, the non-dimensional variables are introduced as follows:

17 $$ \begin{gathered} \xi = \frac{x}{H}{,}\;\zeta = \frac{z}{H}{,}\;\Lambda^{2} = \frac{{\omega^{2} H}}{g}{,}\;\beta = \frac{B}{H}{,}\;\alpha = \frac{h}{H}{,}\;{\mathbf{n}}_{k} = \frac{{{\mathbf{n}}_{k} }}{H}{,}\;\beta_{s} = \frac{{a_{s} }}{H}{,}\; \hfill \\ \lambda_{1n}^{s} = \frac{n\pi }{{{(}\beta_{s + 1} - \beta_{s} {)}}}{,}\left( {s = 0{,}\;1{,}\; \ldots {,}\;M + 1} \right){,}\;\lambda_{2n} = \frac{{{(}2n - 1{)}\pi }}{{2{(}1 - \alpha {)}}} \hfill \\ \end{gathered} $$

For the liquid subdomain $\Omega_{i} \left( {i = s + 1,s = 0,\;1, \ldots ,M} \right)$, one has

18 $$ \begin{gathered} \Phi_{i} \left( {\xi ,\zeta } \right) = A_{i0}^{0} + \sum\limits_{n = 1}^{\infty } {A_{in}^{1} \cosh \lambda_{2n} \left( {\xi - \delta_{1i} \beta_{s} - \delta_{2i} \beta_{s + 1} + \beta } \right)\cos \lambda_{2n} \left( {\zeta - \alpha } \right)}  \\+ \delta_{3i} \sum\limits_{n = 1}^{\infty } {A_{in}^{2} \cosh \lambda_{2n} \left( {\xi - \beta_{s + 1} + \beta } \right)\cos \lambda_{2n} \left( {\zeta - \alpha } \right)} + \sum\limits_{n = 1}^{\infty } {A_{in}^{{l_{1i} }} \cos \lambda_{1n}^{s} \left( {\xi - \beta_{s} + \beta } \right)\cosh \lambda_{1n}^{s} \left( {\zeta - \alpha } \right)}  \\ + A_{i0}^{{l_{2i} }} H\left( {1 - \zeta } \right) + \sum\limits_{n = 1}^{\infty } {A_{in}^{{l_{2i} }} \cos \lambda_{1n}^{s} \left( {\xi - \beta_{s} + \beta } \right)\sinh \lambda_{1n}^{s} \left( {\zeta - 1} \right)} \\ \end{gathered} $$

in which,

19 $$ \delta_{1i} = \left\{ \begin{gathered} 1,\;\;(i \ne M + 1) \hfill \\ 0,\;\;(i = M + 1) \hfill \\ \end{gathered} \right.,\;\delta_{2i} = \left\{ \begin{gathered} 0,\;\;(i \ne M + 1) \hfill \\ 1,\;\;(i = M + 1) \hfill \\ \end{gathered} \right.,\;\left( {\delta_{3i} ,l_{1i} ,l_{2i} } \right) = \left\{ \begin{gathered} \left( {0,2,3} \right),\;\;(i = 1,M + 1) \hfill \\ \left( {1,3,4} \right),\;\;(i = 2,\; \ldots ,\;M) \hfill \\ \end{gathered} \right. $$

For the liquid subdomain $\Omega_{i} \;\left( {i = s + M + 2,s = 0,\;1, \ldots ,M} \right)$, one has

20 $$ \Phi_{i} \left( {\xi ,\zeta } \right) =  A_{i0}^{0} + \sum\limits_{n = 1}^{\infty } {A_{in}^{1} \cos \lambda_{1n}^{s} \left( {\xi - \beta_{s} + \beta } \right)\cosh \lambda_{1n}^{s} \zeta } $$

In Eqs. (18) and (20), $A_{in}^{l} \;\left( {i = 1,\;2, \ldots ,\;2M + 2;\;l = 0,\;1,\;2,\;3,\;4;\;n = 0,\;1,\;2,\;3, \ldots } \right)$ denotes unknown coefficients for the velocity potential components of the corresponding subdomains, which are acquired through artificial interfaces and free surface conditions.

### Eigenfrequency equation

According to $\varphi_{i} (x,\;z,\;t) = {\rm j}\omega {\rm e}^{{{\rm j}\omega t}} \Phi_{i} \left( {x,z} \right)$, the liquid velocity satisfies the sloshing condition of free surface in Eq. (21) and continuity condition of artificial surfaces in Eq. (22):

21 $$ \left. {\frac{{\partial \Phi_{i} }}{\partial \zeta }} \right|_{\zeta = 1} - \Lambda^{2} \left. {\Phi_{i} } \right|_{\zeta = 1} = 0,\;(i = 1,2,\;...,M + 1) $$

22 $$ \Phi_{i} = \Phi_{{i^{\prime}}} ,\;\frac{{\partial \Phi_{i} }}{{\partial {\mathbf{n}}_{k} }} = \frac{{\partial \Phi_{{i^{\prime}}} }}{{\partial {\mathbf{n}}_{k} }},\;{\text{at the surface }}\Gamma_{k} $$

In Eq. (22), the ordered triple (*i*, *i*′, *k*) meets the relation in Eq. (6). Considering the continuity condition at the artificial surface $\Gamma_{k}$ of the two adjacent subdomains $\Omega_{i}$ and $\Omega_{{i^{\prime}}} \;(i = s + 1,\;i{\prime} = s + M + 2,\;k = \, 2s + 1,\;s = 0,\;1, \ldots ,\;M)$, Eqs. (18) and (20) are introduced into Eq. (22) and truncated to *N* terms:

23 $$ \begin{gathered} A_{i0}^{0} + \sum\limits_{n = 1}^{N} {A_{in}^{1} \cosh \lambda_{2n} \left( {\xi - \delta_{1i} \beta_{s} - \delta_{2i} \beta_{s + 1} + \beta } \right)} + \delta_{3i} \sum\limits_{n = 1}^{N} {A_{in}^{2} \cosh \lambda_{2n} \left( {\xi - \beta_{s + 1} + \beta } \right)} \\ + \sum\limits_{n = 1}^{N} {A_{in}^{{l_{1i} }} \cos \lambda_{1n}^{s} \left( {\xi - \beta_{s} + \beta } \right)} - A_{i0}^{{l_{2i} }} H\left( {1 - \alpha } \right) - \sum\limits_{n = 1}^{N} {A_{in}^{{l_{2i} }} \cos \lambda_{1n}^{s} \left( {\xi - \beta_{s} + \beta } \right)\sinh \lambda_{1n}^{s} \left( {\alpha - 1} \right)}  \\ = A_{{i^{\prime}0}}^{0} + \sum\limits_{n = 1}^{N} {A_{{i^{\prime}n}}^{1} \cos \lambda_{1n}^{s} \left( {\xi - \beta_{s} + \beta } \right)\cosh \lambda_{1n}^{s} \alpha } \\ \end{gathered} $$

24 $$ \begin{gathered} - A_{i0}^{{l_{2i} }} H + \sum\limits_{n = 1}^{N} {A_{in}^{{l_{2i} }} \lambda_{1n}^{s} \cos \lambda_{1n}^{s} \left( {\xi - \beta_{s} + \beta } \right)\cosh \lambda_{1n}^{s} \left( {\alpha - 1} \right)}  \\ = \sum\limits_{n = 1}^{N} {A_{{i^{\prime}n}}^{1} \lambda_{1n}^{s} \cos \lambda_{1n}^{s} \left( {\xi - \beta_{s} + \beta } \right)\sinh \lambda_{1n}^{s} \alpha } \\ \end{gathered} $$

Taking the continuity condition at the artificial surface $\Gamma_{k}$ of the two adjacent subdomains $\Omega_{i}$ and $\Omega_{{i^{\prime}}} \;(i = s,\;i{\prime} = s + 1,\;k = \, 2s,\;s = 1,\;2, \ldots ,\;M)$ into account, Eqs. (18) and (20) are introduced into Eq. (22) truncating *n* in the series up to *N*:

25 $$ \begin{gathered} A_{i0}^{0} + \sum\limits_{n = 1}^{N} {A_{in}^{1} \cosh \lambda_{2n} \left( {\beta_{s} - \beta_{s - 1} } \right)\cos \lambda_{2n} \left( {\zeta - \alpha } \right)}  \hfill \\+ \delta_{3i} \sum\limits_{n = 1}^{N} {A_{in}^{2} \cos \lambda_{2n} \left( {\zeta - \alpha } \right)} + \sum\limits_{n = 1}^{N} {A_{in}^{{l_{1i} }} \cos \lambda_{1n}^{s} \left( {\beta_{s} - \beta_{s - 1} } \right)\cosh \lambda_{1n}^{q} \left( {\zeta - \alpha } \right)}  \hfill \\ + A_{i0}^{{l_{2i} }} H\left( {1 - \zeta } \right) + \sum\limits_{n = 1}^{N} {A_{in}^{{l_{2i} }} \cos \lambda_{1n}^{s} \left( {\beta_{s} - \beta_{s - 1} } \right)\sinh \lambda_{1n}^{s} \left( {\zeta - 1} \right)} = A_{{i^{\prime}0}}^{0}  \hfill \\ + \sum\limits_{n = 1}^{N} {A_{{i^{\prime}n}}^{1} \cosh \lambda_{2n} \delta_{{2i^{\prime}}} \left( {\beta_{s} - \beta_{s + 1} } \right)\cos \lambda_{2n} \left( {\zeta - \alpha } \right)}  \hfill \\ + \delta_{{3i^{\prime}}} \sum\limits_{n = 1}^{N} {A_{{i^{\prime}n}}^{2} \cosh \lambda_{2n} \left( {\beta_{s} - \beta_{s + 1} } \right)\cos \lambda_{2n} \left( {\zeta - \alpha } \right)}  \hfill \\ + \sum\limits_{n = 1}^{N} {A_{in}^{{l_{{1i^{\prime}}} }} \cosh \lambda_{1n}^{q} \left( {\zeta - \alpha } \right)} + A_{i0}^{{l_{{2i^{\prime}}} }} H\left( {1 - \zeta } \right) + \sum\limits_{n = 1}^{N} {A_{{i^{\prime}n}}^{{l_{2i} }} \sinh \lambda_{1n}^{s} \left( {\zeta - 1} \right)} \hfill \\ \end{gathered} $$

26 $$ \begin{gathered} \sum\limits_{n = 1}^{N} {A_{in}^{1} \lambda_{2n} \sinh \lambda_{2n} \left( {\beta_{s} - \beta_{s - 1} } \right)\cos \lambda_{2n} \left( {\zeta - \alpha } \right)}  \hfill \\ = \delta_{{2i^{\prime}}} \sum\limits_{n = 1}^{N} {A_{{i^{\prime}n}}^{1} \lambda_{2n} \sinh \lambda_{2n} \left( {\beta_{s} - \beta_{s + 1} } \right)\cos \lambda_{2n} \left( {\zeta - \alpha } \right)}  \hfill \\ + \delta_{{3i^{\prime}}} \sum\limits_{n = 1}^{N} {A_{{i^{\prime}n}}^{2} \lambda_{2n} \sinh \lambda_{2n} \left( {\beta_{s} - \beta_{s + 1} } \right)\cos \lambda_{2n} \left( {\zeta - \alpha } \right)} \hfill \\ \end{gathered} $$

Considering the oscillation condition of the free surface $\Sigma_{i} \;\left( {i = 1,\;2, \ldots ,\;M + 1} \right)$ of subdomain $\Omega_{i}$, introducing Eqs. (18) and (20) into Eq. (21) and truncating *n* in series up to *N* obtain

27 $$ \begin{gathered} - \sum\limits_{n = 1}^{N} {A_{in}^{1} \lambda_{2n} \cosh \lambda_{2n} \left( {\xi - \delta_{1i} \beta_{s} - \delta_{2i} \beta_{s + 1} + \beta } \right)\sin \lambda_{2n} \left( {1 - \alpha } \right)}  \hfill \\ - \delta_{3i} \sum\limits_{n = 1}^{N} {A_{in}^{2} \lambda_{2n} \cosh \lambda_{2n} \left( {\xi - \beta_{s + 1} + \beta } \right)\sin \lambda_{2n} \left( {1 - \alpha } \right)}  \hfill \\ + \sum\limits_{n = 1}^{N} {A_{in}^{{l_{1i} }} \lambda_{1n}^{s} \cos \lambda_{1n}^{s} \left( {\xi - \beta_{s} + \beta } \right)\sinh \lambda_{1n}^{q} \left( {1 - \alpha } \right)}  \hfill \\ - A_{i0}^{{l_{2i} }} H + \sum\limits_{n = 1}^{N} {A_{in}^{{l_{2i} }} \lambda_{1n}^{s} \cos \lambda_{1n}^{s} \left( {\xi - \beta_{s} + \beta } \right)}  \hfill \\ - \Lambda^{2} \left[ {A_{i0}^{0} + \sum\limits_{n = 1}^{N} {A_{in}^{{l_{1i} }} \cos \lambda_{1n}^{s} \left( {\xi - \beta_{s} + \beta } \right)\cosh \lambda_{1n}^{q} \left( {\zeta - \alpha } \right)} } \right] = 0 \hfill \\ \end{gathered} $$

Based on the Fourier series expansion technique, the spatial coordinate $\xi$ can be eliminated by multiplying Eqs. (23), (24) and (27) with $\cos \lambda_{1m}^{s} \left( {\xi - \beta_{s} + \beta } \right)\;({\text{when}}\;m = 0,\;1, \ldots ,\;N)$ and making integral from $\beta_{s} - \beta$ to $\beta_{s + 1} - \beta$; the spatial coordinate $\zeta$ can be eliminated by multiplying Eqs. (25), (26) and (27) with $\cos \lambda_{2n} \left( {\zeta - \alpha } \right)\;({\text{when}}\;m = 1,\;2, \ldots ,\;N)$ and making integral from $\alpha$ to 1. A system of linear eigenfrequency equations for the unknown coefficients $A_{in}^{l}$ can be expressed as the form of

28 $$ \left[ {{\mathbf{M}} - \Lambda^{2} {\mathbf{K}}} \right]\left\{ {\mathbf{A}} \right\} = 0 $$

in which, $\Lambda$ represents nondimensional sloshing frequencies. The coefficient vector {**A**}, generalized mass matrix **M** and stiffness matrix **K** have

29 $$ \left\{ {\mathbf{A}} \right\} = \left[ {\left\{ {A_{1n}^{l} } \right\},\left\{ {A_{2n}^{l} } \right\},\; \ldots ,\left\{ {A_{M + 1n}^{l} } \right\},\left. {\left\{ {A_{M + 2n}^{l} } \right\},\; \ldots ,\left\{ {A_{2M + 2n}^{l} } \right\}} \right]^{T} } \right. $$

30 $$ {\mathbf{M}} = \left[ {\begin{array}{*{20}c} {{\mathbf{M}}_{11} } & {{\mathbf{M}}_{12} } \\ {{\mathbf{M}}_{21} } & {\mathbf{0}} \\ \end{array} } \right],\;{\mathbf{K}} = \left[ {\begin{array}{*{20}c} {\mathbf{0}} & {\mathbf{0}} \\ {{\mathbf{K}}_{21} } & {\mathbf{0}} \\ \end{array} } \right] $$

in which, the detailed forms of the submatrices have

31 $$ {\mathbf{M}}_{11} = diag({\mathbf{J}}_{1}^{11} ,{\mathbf{J}}_{2}^{11} ,\;...,{\mathbf{J}}_{M + 1}^{11} ),\;{\mathbf{M}}_{12} = diag({\mathbf{J}}_{M + 2}^{12} ,{\mathbf{J}}_{M + 3}^{12} ,\;...,{\mathbf{J}}_{2M + 2}^{12} ) $$

32 $$ {\mathbf{M}}_{21} = \left[ {\begin{array}{*{20}c} {{\mathbf{J}}_{1}^{21} } & {{\overline{\mathbf{J}}}_{2}^{21} } & {\mathbf{0}} & \cdots & {\mathbf{0}} \\ {\mathbf{0}} & {{\mathbf{J}}_{2}^{21} } & {{\overline{\mathbf{J}}}_{3}^{21} } & \cdots & {\mathbf{0}} \\ \vdots & \vdots & \ddots & \ddots & \vdots \\ {\mathbf{0}} & {\mathbf{0}} & \cdots & {{\mathbf{J}}_{M}^{21} } & {{\overline{\mathbf{J}}}_{M + 1}^{21} } \\ {{\mathbf{J}}_{1}^{surf} } & {{\mathbf{J}}_{2}^{surf} } & \cdots & {{\mathbf{J}}_{M}^{surf} } & {{\mathbf{J}}_{M + 1}^{surf} } \\ \end{array} } \right],\;{\mathbf{K}}_{21} = \left[ {\begin{array}{*{20}c} {\mathbf{0}} & {\mathbf{0}} & \cdots & {\mathbf{0}} \\ {\mathbf{0}} & {\mathbf{0}} & \cdots & {\mathbf{0}} \\ \vdots & \vdots & \vdots & \vdots \\ {\mathbf{0}} & {\mathbf{0}} & \cdots & {\mathbf{0}} \\ {{\mathbf{G}}_{1}^{surf} } & {{\mathbf{G}}_{2}^{surf} } & \cdots & {{\mathbf{G}}_{M + 1}^{surf} } \\ \end{array} } \right] $$

The natural frequency $\Lambda$ and corresponding eigenvector $A_{in}^{l}$ are acquired by solving eigenfrequency equation Eq. (28) with the utilization of the generalized eigenvalue method. Substituting the coefficient vector into Eqs. (18) and (20) obtains the sloshing mode of the free surface:

33 $$ S = \left. \Phi \right|_{\zeta = 0} $$

## Forced vibration of liquid

### Governing equations and boundary conditions

The velocity potential $\varphi_{i} (x,z,t)$ for each subdomain meets Laplace equation:

34 $$ \nabla^{2} \varphi_{i} = 0,\;(x,\;z) \in \Omega_{i} $$

Since the storage container is undergoing horizontal ground motion $u(t)$, $\varphi_{i} (x,z,t)$ should meet impermeability boundary conditions of the wall, bottom and baffles of the container:

35 $$ \frac{{\partial \varphi_{i} }}{{\partial {\overline{\mathbf{n}}}}} = \dot{u}(t),\;\frac{{\partial \varphi_{i} }}{{\partial {\tilde{\mathbf{n}}}}} = 0 $$

in which ${\overline{\mathbf{n}}}$ and ${\tilde{\mathbf{n}}}$ are unit outer normal vectors of vertical and horizontal surfaces, respectively. Besides, $\varphi_{i} (x,z,t)$ meets the boundary condition of free surface $\Sigma_{i}$:

36 $$ \left. {\frac{{\partial \varphi_{i} }}{\partial t}} \right|_{z = H} + g\eta_{i} = 0,\;(i = 1,\;2, \ldots ,\;M + 1) $$

where the sloshing height function $\eta_{i}$ of free surface for $\Omega_{i}$ has the form of

37 $$ \eta_{i} = \int_{0}^{t} {\left. {\frac{{\partial \varphi_{i} }}{\partial z}} \right|_{z = H} {\rm d}t} $$

At the artificial surface $\Gamma_{k}$ of the two adjacent liquid subdomains $\Omega_{i}$ and $\Omega_{{i^{\prime}}}$, $\varphi_{i} (x,z,t)$ meet the pressure and velocity continuity conditions as follows:

38 $$ \frac{{\partial \varphi_{i} }}{\partial t} = \frac{{\partial \varphi_{{i^{\prime}}} }}{\partial t},\;\frac{{\partial \varphi_{i} }}{{\partial {\mathbf{n}}_{k} }} = \frac{{\partial \varphi_{{i^{\prime}}} }}{{\partial {\mathbf{n}}_{k} }} $$

Besides, $\varphi_{i} (x,z,t)$ should satisfy initial conditions of motion:

39 $$ \left. {\varphi_{i} } \right|_{t = 0} = \varphi_{0} ,\;\left. {\dot{\varphi }_{i} } \right|_{t = 0} = \dot{\varphi }_{0} $$

### Solution to liquid velocity potential

The liquid velocity potential $\varphi_{i} (x,z,t)$ can be composed of the two parts: the first one is the impulsive velocity potential $\varphi_{i}^{{\rm I}} (x,z,t)$ for which the liquid shows like an attached rigid mass moving synchronously with the storage container; the second one is the convective velocity potential $\varphi_{i}^{{\rm C}} (x,z,t)$ for which liquid shows like a series of attached elastic masses undergoing liquid sloshing at the free surface, namely, $\varphi_{i} (x,z,t) = \varphi_{i}^{{\rm I}} (x,z,t) + \varphi_{i}^{{\rm C}} (x,z,t).$ Thus, Eqs. (34)–(39) can be displayed by

40 $$ \nabla^{2} \varphi_{i}^{{\text{I}}} = 0,\;\nabla^{2} \varphi_{i}^{{\rm C}} = 0,\;(x,z) \in \Omega_{i} $$

41 $$ \frac{{\partial \varphi_{i}^{{\rm I}} }}{{\partial {\overline{\mathbf{n}}}}} = \dot{u}(t),\;\frac{{\partial \varphi_{i}^{{\rm I}} }}{{\partial {\tilde{\mathbf{n}}}}} = 0,\;\frac{{\partial \varphi_{i}^{{\rm C}} }}{{\partial {\overline{\mathbf{n}}}}} = 0,\;\frac{{\partial \varphi_{i}^{{\rm C}} }}{{\partial {\tilde{\mathbf{n}}}}} = 0 $$

42 $$ \left. {\frac{{\partial \varphi_{i}^{{\rm C}} }}{\partial t}} \right|_{z = H} + g\eta_{i}^{{\rm C}} = - \left. {\frac{{\partial \varphi_{i}^{{\rm I}} }}{\partial t}} \right|_{z = H} - g\eta_{i}^{{\rm I}} $$

43 $$ \eta_{i}^{{\rm I}} = \int_{0}^{t} {\left. {\frac{{\partial \varphi_{i}^{{\rm I}} }}{\partial z}} \right|_{z = H} {\rm d}t} ,\;\eta_{i}^{{\rm C}} = \int_{0}^{t} {\left. {\frac{{\partial \varphi_{i}^{{\rm C}} }}{\partial z}} \right|_{z = H} {\rm d}t} $$

44 $$ \frac{{\partial \varphi_{i}^{{\rm I}} }}{\partial t} = \frac{{\partial \varphi_{{i^{\prime}}}^{{\rm I}} }}{\partial t},\;\frac{{\partial \varphi_{i}^{{\rm I}} }}{{\partial {\mathbf{n}}_{k} }} = \frac{{\partial \varphi_{{i^{\prime}}}^{{\rm I}} }}{{\partial {\mathbf{n}}_{k} }},\;\frac{{\partial \varphi_{i}^{{\rm C}} }}{\partial t} = \frac{{\partial \varphi_{{i^{\prime}}}^{{\rm C}} }}{\partial t},\;\frac{{\partial \varphi_{i}^{{\rm C}} }}{{\partial {\mathbf{n}}_{k} }} = \frac{{\partial \varphi_{{i^{\prime}}}^{{\rm C}} }}{{\partial {\mathbf{n}}_{k} }} $$

in which, $\eta_{i}^{{\text{I}}}$ and $\eta_{i}^{{\text{C}}}$ represent, respectively, sloshing height functions corresponding to impulsive and convective velocity potentials. Based on governing motion equation in Eq. (40), the rigid boundary condition in Eq. (41) and the artificial interface condition in Eq. (44) that $\varphi_{i}^{{\rm I}} (x,z,t)$ satisfies, one has

45 $$ \varphi_{i}^{{\rm I}} (x,z,t) = x\dot{u}(t),\;(x,z) \in \Omega_{i} $$

Introducing Eqs. (43) and (45) into Eq. (42), $\varphi_{i}^{{\rm C}} (x,z,t)$ meets boundary condition for free surface:

46 $$ \left. {\frac{{\partial \varphi_{i}^{{\rm C}} }}{\partial t}} \right|_{z = H} + g\eta_{i}^{{\rm C}} = - x\ddot{u}(t) $$

Through introducing the generalized coordinate $q_{n} (t)$, $\varphi_{i}^{{\rm C}} (x,z,t)$ is expanded to series form of vibration modes on the basis of the superposition technique:

47 $$ \varphi_{i}^{{\rm C}} (x,z,t) = \sum\limits_{n = 1}^{\infty } {\dot{q}_{n} (t)\Phi_{in} (x,z)} ,\;(i = 1,\;2, \ldots ,\;2M + 2) $$

in which the *n*th sloshing mode $\Phi_{in} (x,z)$ for $\Omega_{i}$ can be acquired by the eigenvalue problem in Subsection 3.2.

### Orthogonality of coupling mode shapes

Consider the sloshing frequencies $\omega_{m}$ and $\omega_{n} \;(\omega_{m} \ne \omega_{n} )$ corresponding to the sloshing modes $\Phi_{im}$ and $\Phi_{in}$, respectively. According to the Green formula, one can write

48 $$ \iint_{{\Omega_{i} }} {\nabla \Phi_{im} \cdot \nabla \Phi_{in} { + }\Phi_{im} \nabla^{2} \Phi_{in} {\rm d\Omega }} = \int_{{L_{i} }} {\Phi_{im} \frac{{\Phi_{in} }}{{\partial {\mathbf{\overset{\lower0.5em\hbox{$\smash{\scriptscriptstyle\frown}$}}{n} }}}}{\rm d}S} $$

in which $${\mathbf{\overset{\lower0.5em\hbox{$\smash{\scriptscriptstyle\frown}$}}{n} }}$$ denotes the unit tangent vector of the boundary curve $$L_{i}$$ for the subdomain $$\Omega_{i}$$. Then, for all the liquid subdomains $$\Omega_{i} \;(i = 1,\;2, \ldots ,\;2M + 2)$$, adding both sides of Eq. (48) gives

49 $$ \sum\limits_{i = 1}^{2M + 2} {\iint_{{\Omega_{i} }} {\nabla \Phi_{im} \cdot \nabla \Phi_{in} { + }\Phi_{im} \nabla^{2} \Phi_{in} {\rm d\Omega }}} = \sum\limits_{i = 1}^{2M + 2} {\int_{{L_{i} }} {\Phi_{im} \frac{{\Phi_{in} }}{{\partial {\mathbf{\overset{\lower0.5em\hbox{$\smash{\scriptscriptstyle\frown}$}}{n} }}}}{\rm d}S} } $$

According to governing movement equation and rigid boundary conditions in Eqs. (8)-(10) as well as the continuity condition of artificial interfaces in Eq. (22) that the sloshing modes $\Phi_{im}$ and $\Phi_{in}$ should satisfy, Eq. (49) can be expressed as the form of

50 $$ \sum\limits_{i = 1}^{2M + 2} {\iint_{{\Omega_{i} }} {\nabla \Phi_{im} \cdot \nabla \Phi_{in} {\rm d\Omega }}} = \sum\limits_{i = 1}^{M + 1} {\int_{{\Sigma_{i} }} {\Phi_{im} \frac{{\Phi_{in} }}{{\partial {\mathbf{\overset{\lower0.5em\hbox{$\smash{\scriptscriptstyle\frown}$}}{n} }}}}{\rm d}S} } $$

Similarly, one can also obtain

51 $$ \sum\limits_{i = 1}^{2M + 2} {\iint_{{\Omega_{i} }} {\nabla \Phi_{in} \cdot \nabla \Phi_{im} {\rm d\Omega }}} = \sum\limits_{i = 1}^{M + 1} {\int_{{\Sigma_{i} }} {\Phi_{in} \frac{{\Phi_{im} }}{{\partial {\mathbf{\overset{\lower0.5em\hbox{$\smash{\scriptscriptstyle\frown}$}}{n} }}}}{\rm d}S} } $$

Combining Eqs. (50) and (51) and taking the scalar form yield

52 $$ \sum\limits_{i = 1}^{M + 1} {\int_{{\Sigma_{i} }} {\Phi_{im} \frac{{\Phi_{in} }}{\partial z}{\rm d}S} } = \sum\limits_{i = 1}^{M + 1} {\int_{{\Sigma_{i} }} {\Phi_{in} \frac{{\Phi_{im} }}{\partial z}{\rm d}S} } $$

Introducing the sloshing condition of the free surface into Eq. (52), one has

53 $$ \left( {\omega_{m}^{2} - \omega_{n}^{2} } \right)\sum\limits_{i = 1}^{M + 1} {\int_{{\Sigma_{i} }} {\Phi_{im} \Phi_{in} {\rm d}S} } = 0 $$

Due to $\omega_{m} \ne \omega_{n}$, one can acquire the orthogonality characteristics of the coupling mode shapes:

54 $$ \sum\limits_{i = 1}^{M + 1} {\int_{{\Sigma_{i} }} {\Phi_{im} \Phi_{in} {\rm d}S} } = 0 $$

## Equivalent model of sloshing

Substituting Eqs. (43) and (47) into Eq. (46) yields

55 $$ \sum\limits_{n = 1}^{\infty } {\ddot{q}_{n} (t)\left. {\Phi_{in} (x,z)} \right|_{z = H} } + g\sum\limits_{n = 0}^{\infty } {\left. {q_{n} (t)\frac{{\partial \Phi_{in} (x,z)}}{\partial z}} \right|_{z = H} } = - x\ddot{u}(t) $$

Through multiplying both sides of Eq. (55) by $\left. {\Phi_{im} (x,z)} \right|_{z = H} \;\left( {m = 1,\;2, \ldots } \right)$ and integrating from –*B* to *B*, the spatial coordinate *x* is eliminated. On the basis of the sloshing condition in Eq. (21) and mode orthogonality in Eq. (54), the sloshing response equation can be obtained about $q_{n} (t)$:

56 $$ M_{n} \ddot{q}_{n} (t) + K_{n} q_{n} (t) = - \ddot{u}(t) $$

in which $M_{n}$ and $K_{n}$ represent the generalized modal mass and generalized modal stiffness corresponding to the *n*th vibration mode, respectively, and have the forms of

57 $$ M_{n} = {{\sum\limits_{i = 1}^{{M{ + 1}}} {\int_{{\Sigma_{i} }} {\left[ {\left. {\Phi_{in} (x,z)} \right|_{z = H} } \right]^{2} {\rm d}S} } } \mathord{\left/ {\vphantom {{\sum\limits_{i = 1}^{{M{ + 1}}} {\int_{{\Sigma_{i} }} {\left[ {\left. {\Phi_{in} (x,z)} \right|_{z = H} } \right]^{2} {\rm d}S} } } {\sum\limits_{i = 1}^{M + 1} {\int_{{\Sigma_{i} }} {x\left. {\Phi_{in} (x,z)} \right|_{z = H} {\rm d}S} } }}} \right. \kern-0pt} {\sum\limits_{i = 1}^{M + 1} {\int_{{\Sigma_{i} }} {x\left. {\Phi_{in} (x,z)} \right|_{z = H} {\rm d}S} } }} $$

58 $$ K_{n} = \omega_{n}^{2} M_{n} $$

where $\omega_{n}$ denotes the *n*th sloshing frequency for liquid of the container-liquid-baffle coupling system and can be obtained by the eigenvalue equation in “Eigenfrequency equation”.

Based on $\varphi_{i} = \varphi_{i}^{{\rm I}} + \varphi_{i}^{{\rm C}} ,$ the surface sloshing wave height yields

59 $$ \eta_{i} (x,t) = - \frac{1}{g}\left. {\frac{{\partial \varphi_{i} }}{\partial t}} \right|_{z = H} = - \frac{1}{g}\left( {x\ddot{u}(t) + \sum\limits_{n = 1}^{\infty } {\ddot{q}_{n} (t)} \left. {\Phi_{in} (x,z)} \right|_{z = H} } \right),\;(i = 1,\;2,...,\;M + 1) $$

The hydrodynamic pressures owing to sloshing motion are acquired according to Bernoulli equation:

60 $$ P_{i} (x,z,t) = - \rho \frac{{\partial \varphi_{i} }}{\partial t} = - \rho \left( {x\ddot{u}(t) + \sum\limits_{n = 1}^{\infty } {\ddot{q}_{n} (t)\Phi_{in} (x,z)} } \right),\;(i = 1,\;2,...,\;2M + 2) $$

$\rho$ is the liquid density. Through integrating hydrodynamic pressures over surfaces of rigid wall, the hydrodynamic shear can be obtained:

61 $$ F_{x} (t) = \int_{h}^{H} {P_{M + 1} (B,z,t) - P_{1} ( - B,z,t)} {\rm d}z + \sum\limits_{m = 1}^{M + 1} {\int_{0}^{h} {P_{m + M + 1} (a_{m} - B,z,t) - P_{m + M + 1} (a_{m - 1} - B,z,t)} {\rm d}z} $$

Similarly, the hydrodynamic overturning moment exerting on surfaces about *y* axis has

62 $$ M_{y} (t) = M_{{{\rm wall}}} (t) + M_{{{\rm bottom}}} (t) + M_{{{\rm baffle}}} (t) $$

where $M_{{{\rm wall}}} (t)$, $M_{{{\rm bottom}}} (t)$ and $M_{{{\rm baffle}}} (t)$ denote hydrodynamic overturning moments exerting on the rigid container wall, container bottom and vertical baffles, respectively. The expressions are in the form of

63 $$ M_{{{\rm wall}}} (t) = \int_{h}^{H} {\left[ {P_{M + 1} (B,z,t) - P_{1} ( - B,z,t)} \right]} z{\rm d}z + \int_{0}^{h} {\left[ {P_{2M + 2} (B,z,t) - P_{M + 2} ( - B,z,t)} \right]} z{\rm d}z $$

64 $$ M_{{{\text{bottom}}}} (t) = \sum\limits_{m = 1}^{M + 1} {\int_{{a_{m - 1} - B}}^{{a_{m} - B}} {P_{m + M + 1} (x,0,t)} x{\rm d}x} $$

65 $$ M_{{{\text{baffle}}}} (t) = \sum\limits_{m = 1}^{M} {\int_{0}^{h} {\left[ {P_{m + M + 1} (a_{m} - B,z,t) - P_{m + M + 2} (a_{m} - B,z,t)} \right]} z{\rm d}z} $$

Combined with the Eqs. (60)-(65), one can obtain

66 $$ F(t) = - \sum\limits_{n = 1}^{\infty } {\ddot{q}_{n} (t)} A_{n} - 2\rho BH\ddot{u}(t) $$

67 $$ M_{{{\rm wall}}} (t) = - \sum\limits_{n = 1}^{\infty } {\ddot{q}_{n} (t)} B_{n} - \rho BH^{2} \ddot{u}(t) $$

68 $$ M_{{{\rm bottom}}} (t) = - \sum\limits_{n = 1}^{\infty } {\ddot{q}_{n} (t)} C_{n} - \frac{2}{3}\rho B^{3} \ddot{u}(t) $$

69 $$ M_{{{\rm baffle}}} (t) = - \sum\limits_{n = 1}^{\infty } {\ddot{q}_{n} (t)} D_{n} $$

in which,

70 $$ A_{n} = \rho \int_{h}^{H} {\Phi_{M + 1,n} } (B,z) - \Phi_{1,n} ( - B,z)dz + \rho \sum\limits_{m = 1}^{M + 1} {\int_{0}^{h} {\Phi_{m + M + 1,n} (a_{m} - B,z) - } } \Phi_{m + M + 1,n} (a_{m - 1} - B,z)dz $$

71 $$ B_{n} = \rho \int_{h}^{H} {\left[ {\Phi_{M + 1,n} (B,z) - \Phi_{1,n} ( - B,z)} \right]z} dz + \rho \int_{0}^{h} {\left[ {\Phi_{2M + 2,n} (B,z) - \Phi_{M + 2,n} ( - B,z)} \right]z} dz $$

72 $$ C_{n} = \rho \sum\limits_{m = 1}^{M + 1} {\int_{{a_{m - 1} - B}}^{{a_{m} - B}} {\Phi_{m + M + 1} (x,0)xdx} } $$

73 $$ D_{n} = \rho \sum\limits_{m = 1}^{M} {\int_{0}^{h} {\left[ {\Phi_{m + M + 1,n} (a_{m} - B,z) - \Phi_{m + M + 2,n} (a_{m} - B,z)} \right]z} dz} $$

**Figure 3:**
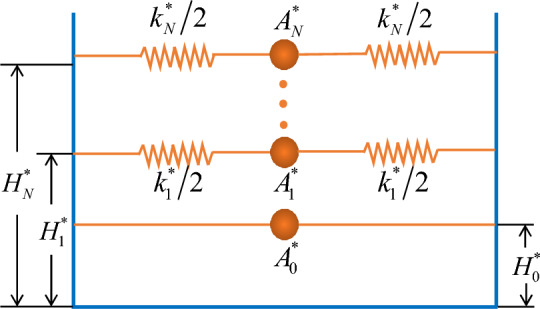
The equivalent mechanical model for sloshing.

Taking $\ddot{q}_{n}^{*} (t) = M_{n} \ddot{q}_{n} (t)$ and $q_{n}^{*} (t) = M_{n} q_{n} (t)$, Eq. (56) has

74 $$ A_{n}^{*} \ddot{q}_{n}^{*} (t) + k_{n}^{*} q_{n}^{*} (t) = - A_{n}^{*} \ddot{u}(t) $$

in which $A_{n}^{*} \;(A_{n}^{*} = {{A_{n} } \mathord{\left/ {\vphantom {{A_{n} } {M_{n} }}} \right. \kern-0pt} {M_{n} }})$ and $k_{n}^{*}$ denote, respectively, the convective mass and corresponding spring stiffness for the proposed equivalent model. $\ddot{q}_{n}^{*} (t)$ is the relative acceleration for each convective mass oscillator. Introducing $\ddot{q}_{n}^{{}} (t) = {{\ddot{q}_{n}^{*} (t)} \mathord{\left/ {\vphantom {{\ddot{q}_{n}^{*} (t)} {M_{n} }}} \right. \kern-0pt} {M_{n} }}$ into Eq. (59) and truncating series, one has

75 $$ \eta_{i} (x,t) = - \frac{1}{g}\left( {x\ddot{u}(t) + \sum\limits_{n = 1}^{\infty } {\frac{{\ddot{q}_{n}^{*} (t)}}{{M_{n} }}} \left. {\Phi_{in} (x,z)} \right|_{z = H} } \right),\;(i = 1,\;2,...,\;M + 1) $$

76 $$ F(t) = - \sum\limits_{n = 1}^{N} {\left[ {\ddot{q}_{n}^{*} + \ddot{u}(t)} \right]} A_{n}^{*} - \left( {2\rho BH - \sum\limits_{n = 1}^{N} {A_{n}^{*} } } \right)\ddot{u}(t) $$

77 $$ M_{{{\rm wall}}} (t) = - \sum\limits_{n = 1}^{N} {\left[ {\ddot{q}_{n}^{*} + \ddot{u}(t)} \right]} B_{n}^{*} - \left( {\rho BH^{2} - \sum\limits_{n = 1}^{N} {B_{n}^{*} } } \right)\ddot{u}(t) $$

78 $$ M_{{{\rm bottom}}} (t) = - \sum\limits_{n = 1}^{N} {\left[ {\ddot{q}_{n}^{*} + \ddot{u}(t)} \right]} C_{n}^{*} - \left( {\frac{2}{3}\rho B^{3} - \sum\limits_{n = 1}^{N} {C_{n}^{*} } } \right)\ddot{u}(t) $$

79 $$ M_{{{\rm baffle}}} (t) = - \sum\limits_{n = 1}^{N} {\left[ {\ddot{q}_{n}^{*} + \ddot{u}(t)} \right]} D_{n}^{*} - \left( { - \sum\limits_{n = 1}^{N} {D_{n}^{*} } } \right)\ddot{u}(t) $$

where $B_{n}^{*} = {{B_{n} } \mathord{\left/ {\vphantom {{B_{n} } {M_{n} }}} \right. \kern-0pt} {M_{n} }},\;C_{n}^{*} = {{C_{n} } \mathord{\left/ {\vphantom {{C_{n} } {M_{n} }}} \right. \kern-0pt} {M_{n} }}$ and $D_{n}^{*} = {{D_{n} } \mathord{\left/ {\vphantom {{D_{n} } {M_{n} }}} \right. \kern-0pt} {M_{n} }}.$ According to Eqs. (75)–(79) and through producing the same hydrodynamic shear and moment as those obtained from the original container-liquid-baffle system, an equivalent analytical model of sloshing in a rectangular baffled container undergoing arbitrary horizontal excitation is constructed in Fig. 3, where $A_{0}^{*}$ is the impulsive mass; $H_{n}^{*}$ and $H_{0}^{*}$ are, respectively, corresponding heights of equivalent masses.

## Determination of model parameters

Taking hydrodynamic moments exerting on the container wall, container bottom and vertical baffles into consideration, model mechanical parameters can be obtained from Table 1. The equivalent heights of convective mass oscillators are given as follows when only taking the hydrodynamic moment exerting on the container wall into account:

80 $$ H_{n}^{*} = {{B_{n}^{*} } \mathord{\left/ {\vphantom {{B_{n}^{*} } {A_{n}^{*} }}} \right. \kern-0pt} {A_{n}^{*} }},\;\;\;H_{0}^{*} = {{\left( {\rho BH^{2} - \sum\limits_{n = 1}^{N} {B_{n}^{*} } } \right)} \mathord{\left/ {\vphantom {{\left( {\rho BH^{2} - \sum\limits_{n = 1}^{N} {B_{n}^{*} } } \right)} {\left( {2\rho BH - \sum\limits_{n = 1}^{N} {A_{n}^{*} } } \right)}}} \right. \kern-0pt} {\left( {2\rho BH - \sum\limits_{n = 1}^{N} {A_{n}^{*} } } \right)}} $$

**Table 1:** The parameters in the proposed mass-spring analytical model.

Parameters	Detailed expressions
$A_{n}^{*}$	${{A_{n} } \mathord{\left/ {\vphantom {{A_{n} } {M_{n} }}} \right. \kern-0pt} {M_{n} }}$
$A_{0}^{*}$	$2\rho BH - \sum\limits_{n = 1}^{N} {A_{n}^{*} }$
$H_{n}^{*}$	$\frac{{B_{n}^{*} + C_{n}^{*} + D_{n}^{*} }}{{A_{n}^{*} }}$
$H_{0}^{*}$	$\frac{{\left( {\rho BH^{2} - \sum\limits_{n = 1}^{N} {B_{n}^{*} } } \right) + \left( {\frac{2}{3}\rho B^{3} - \sum\limits_{n = 1}^{N} {C_{n}^{*} } } \right) + \left( { - \sum\limits_{n = 1}^{N} {D_{n}^{*} } } \right)}}{{2\rho BH - \sum\limits_{n = 1}^{N} {A_{n}^{*} } }}$
$k_{n}^{*}$	$\omega_{n}^{2} A_{n}^{*}$

Taking hydrodynamic moments exerting on the container wall and vertical baffles into account, one has

81 $$ H_{n}^{*} = {{\left( {B_{n}^{*} + D_{n}^{*} } \right)} \mathord{\left/ {\vphantom {{\left( {B_{n}^{*} + D_{n}^{*} } \right)} {A_{n}^{*} }}} \right. \kern-0pt} {A_{n}^{*} }},\;\;\;H_{0}^{*} = {{\left( {\rho BH^{2} - \sum\limits_{n = 1}^{N} {B_{n}^{*} } - \sum\limits_{n = 1}^{N} {D_{n}^{*} } } \right)} \mathord{\left/ {\vphantom {{\left( {\rho BH^{2} - \sum\limits_{n = 1}^{N} {B_{n}^{*} } - \sum\limits_{n = 1}^{N} {D_{n}^{*} } } \right)} {\left( {2\rho BH - \sum\limits_{n = 1}^{N} {A_{n}^{*} } } \right)}}} \right. \kern-0pt} {\left( {2\rho BH - \sum\limits_{n = 1}^{N} {A_{n}^{*} } } \right)}} $$

According to the proposed mechanical model in Fig. 3, the hydrodynamic shear $F_{x}$ and hydrodynamic moment $M_{y}$ can be given as

82 $$ F_{x} (t) = \sum\limits_{n = 1}^{N} {\left[ {\ddot{q}_{n}^{*} (t) + \ddot{u}(t)} \right]} A_{n}^{*} + A_{0}^{*} \ddot{u}(t) $$

83 $$ M_{y} (t) = \sum\limits_{n = 1}^{N} {\left[ {\ddot{q}_{n}^{*} (t) + \ddot{u}(t)} \right]} A_{n}^{*} H_{n}^{*} + A_{0}^{*} H_{0}^{*} \ddot{u}(t) $$

Define normalized convective mass $\alpha_{n}^{*}$, corresponding spring stiffness $\kappa_{n}^{*}$ and impulsive mass $\alpha_{0}^{*}$ as $\alpha_{n}^{*} = {{A_{n}^{*} } \mathord{\left/ {\vphantom {{A_{n}^{*} } {M_{{\text{f}}} }}} \right. \kern-0pt} {M_{{\text{f}}} }},$ $\kappa_{n}^{*} = {{\alpha_{n}^{*} \omega_{n}^{2} H} \mathord{\left/ {\vphantom {{\alpha_{n}^{*} \omega_{n}^{2} H} g}} \right. \kern-0pt} g}$ and $\alpha_{0}^{*} = {{A_{0}^{*} } \mathord{\left/ {\vphantom {{A_{0}^{*} } {M_{{\text{f}}} }}} \right. \kern-0pt} {M_{{\text{f}}} }}.$ $M_{{\text{f}}}$ denotes the liquid mass. The liquid density is $\rho = 1000\;{\text{kg/m}}^{{3}} .$ Table 2 presents first four convective masses $\alpha_{n}^{*} \;\left( {n = 1,\;2,\;3,\;4} \right)$ and $\alpha_{0}^{*}$ with different baffle parameters for *M* = 2 and ${H \mathord{\left/ {\vphantom {H B}} \right. \kern-0pt} B} = 2$. The two vertical baffles are located symmetrically about the container bottom center. It is found that the first order convective mass oscillator and the impulsive mass occupy great proportion of convective masses and liquid mass, respectively.

**Table 2:** The first four order convective masses $\alpha_{n}^{*} \;\left( {n = 1,\;2,\;3,\;4} \right)$ and impulsive mass $\alpha_{0}^{*}$ with different baffle positions ${{a_{1} } \mathord{\left/ {\vphantom {{a_{1} } B}} \right. \kern-0pt} B},\;{{a_{2} } \mathord{\left/ {\vphantom {{a_{2} } B}} \right. \kern-0pt} B}$ and baffle heights ${h \mathord{\left/ {\vphantom {h H}} \right. \kern-0pt} H}$ for *M* = 2.

$({{a_{1} } \mathord{\left/ {\vphantom {{a_{1} } B}} \right. \kern-0pt} B},\;{{a_{2} } \mathord{\left/ {\vphantom {{a_{2} } B}} \right. \kern-0pt} B},\;{h \mathord{\left/ {\vphantom {h H}} \right. \kern-0pt} H})$	$\alpha_{1}^{*}$	$\alpha_{2}^{*}$	$\alpha_{3}^{*}$	$\alpha_{4}^{*}$	$\alpha_{0}^{*}$
(0.4, 1.6, 0.8)	0.2566	0.0091	0.0021	0.0010	0.7304
(0.4, 1.6, 0.5)	0.2493	0.0092	0.0021	0.0008	0.7377
(0.6, 1.4, 0.2)	0.1837	0.0078	0.0026	0.0007	0.8044
(0.8, 1.2, 0.8)	0.2554	0.0099	0.0021	0.0006	0.7311

**Figure 4:**
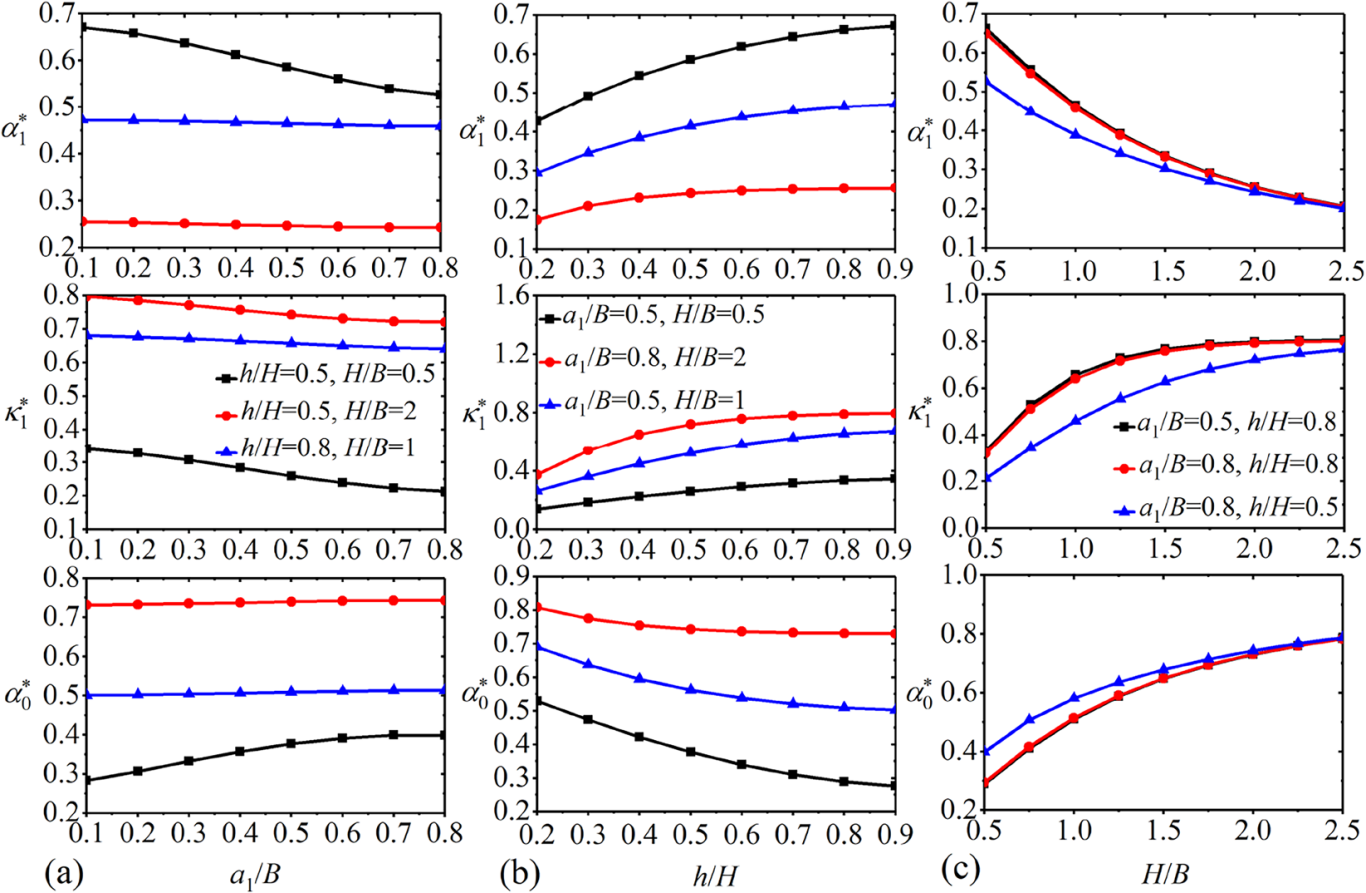
The first order convective mass $\alpha_{1}^{*}$, corresponding spring stiffness $\kappa_{1}^{*}$ and impulsive mass $\alpha_{0}^{*}$ for *M* = 2: (**a**) under non-dimensional baffle positions ${{a_{1} } \mathord{\left/ {\vphantom {{a_{1} } B}} \right. \kern-0pt} B}$; (**b**) under non-dimensional baffle heights ${h \mathord{\left/ {\vphantom {h H}} \right. \kern-0pt} H}$; (**c**) under non-dimensional liquid heights ${H \mathord{\left/ {\vphantom {H B}} \right. \kern-0pt} B}$.

The first order convective mass $\alpha_{1}^{*}$, corresponding spring stiffness $\kappa_{1}^{*}$ and impulsive mass $\alpha_{0}^{*}$ versus various baffle positions ${{a_{1} } \mathord{\left/ {\vphantom {{a_{1} } B}} \right. \kern-0pt} B}$ with *M* = 2, ${h \mathord{\left/ {\vphantom {h {H = 0.5,\;0.8}}} \right. \kern-0pt} {H = 0.5,\;0.8}}$ and ${H \mathord{\left/ {\vphantom {H B}} \right. \kern-0pt} B} = 0.5,\;1.0,\;2.0$ are depicted in Fig. 4a. One can find that as the nondimensional baffle position ${{a_{1} } \mathord{\left/ {\vphantom {{a_{1} } B}} \right. \kern-0pt} B}$ increases, $\alpha_{1}^{*}$ and $\kappa_{1}^{*}$ both decline but $\alpha_{0}^{*}$ increases. Figure 4b shows the results of $\alpha_{1}^{*}$, $\kappa_{1}^{*}$ and $\alpha_{0}^{*}$ for various baffle heights ${h \mathord{\left/ {\vphantom {h H}} \right. \kern-0pt} H}$ with *M* = 2, ${{a_{1} } \mathord{\left/ {\vphantom {{a_{1} } B}} \right. \kern-0pt} B} = 0.5,\;0.8$ and ${H \mathord{\left/ {\vphantom {H B}} \right. \kern-0pt} B} = 0.5,\;1.0,\;2.0$. It is clear that as the vertical baffle approaches the free surface, $\alpha_{1}^{*}$ and $\kappa_{1}^{*}$ both increase whereas $\alpha_{0}^{*}$ decreases. Figure 4c illustrates the variations of $\alpha_{1}^{*}$, $\kappa_{1}^{*}$ and $\alpha_{0}^{*}$ for various liquid heights ${H \mathord{\left/ {\vphantom {H B}} \right. \kern-0pt} B}$ with *M* = 2, ${{a_{1} } \mathord{\left/ {\vphantom {{a_{1} } B}} \right. \kern-0pt} B} = 0.5,\;0.8$ and ${h \mathord{\left/ {\vphantom {h {H = 0.5,\;0.8}}} \right. \kern-0pt} {H = 0.5,\;0.8}}$. It is of interest to note that the greater liquid height implies smaller $\alpha_{1}^{*}$ and larger $\alpha_{0}^{*}$. In addition, the calculation results of $\alpha_{1}^{*}$, $\kappa_{1}^{*}$ and $\alpha_{0}^{*}$ for four combinations of ${{a_{1} } \mathord{\left/ {\vphantom {{a_{1} } B}} \right. \kern-0pt} B}$, ${h \mathord{\left/ {\vphantom {h H}} \right. \kern-0pt} H}$ and ${H \mathord{\left/ {\vphantom {H B}} \right. \kern-0pt} B}$ with $M = 1,\;2$ are listed in Table 3.

**Table 3:** The first order convective mass $\alpha_{1}^{*}$, corresponding spring stiffness $\kappa_{1}^{*}$ and impulsive mass $\alpha_{0}^{*}$ under the baffle position ${{a_{1} } \mathord{\left/ {\vphantom {{a_{1} } B}} \right. \kern-0pt} B}$, baffle height ${h \mathord{\left/ {\vphantom {h H}} \right. \kern-0pt} H}$ and liquid height ${H \mathord{\left/ {\vphantom {H B}} \right. \kern-0pt} B}$ for the baffle number *M* = 1, 2.

*M*	$\left( {{{a_{1} } \mathord{\left/ {\vphantom {{a_{1} } B}} \right. \kern-0pt} B},\;{h \mathord{\left/ {\vphantom {h H}} \right. \kern-0pt} H},\;{H \mathord{\left/ {\vphantom {H B}} \right. \kern-0pt} B}} \right)$	$\alpha_{1}^{*}$	$\kappa_{1}^{*}$	$\alpha_{0}^{*}$
1	(0.2, 0.3, 1.0)	0.4523	0.6215	0.5258
	(0.4, 0.5, 1.5)	0.3274	0.7338	0.6553
	(0.6, 0.7, 2.0)	0.2555	0.7939	0.7313
	(0.8, 0.9, 2.5)	0.2059	0.8051	0.7832
2	(0.2, 0.3, 1.0)	0.4356	0.5737	0.5500
	(0.4, 0.5, 1.5)	0.3186	0.6944	0.6647
	(0.6, 0.7, 2.0)	0.2542	0.7855	0.7326
	(0.8, 0.9, 2.5)	0.2056	0.8010	0.7833

Similarly, the data for $M = 3,\;4,\;5, \ldots$ and $n = 2,\;3,\;4 \ldots$ could be obtained in figures and tables. Due to the length limitation of the paper, these data are not given in the present investigation. By referring to the data such as given in Tables 2 and 3 as well as Fig. 4, the calculation values of mechanical parameters of the present model can be directly obtained. By substituting these parameter values into Eq. (74), the liquid responses could be easily acquired via the Newmark-*β* approach.

## Numerical examples

### Sloshing frequency and mode

**Figure 5:**
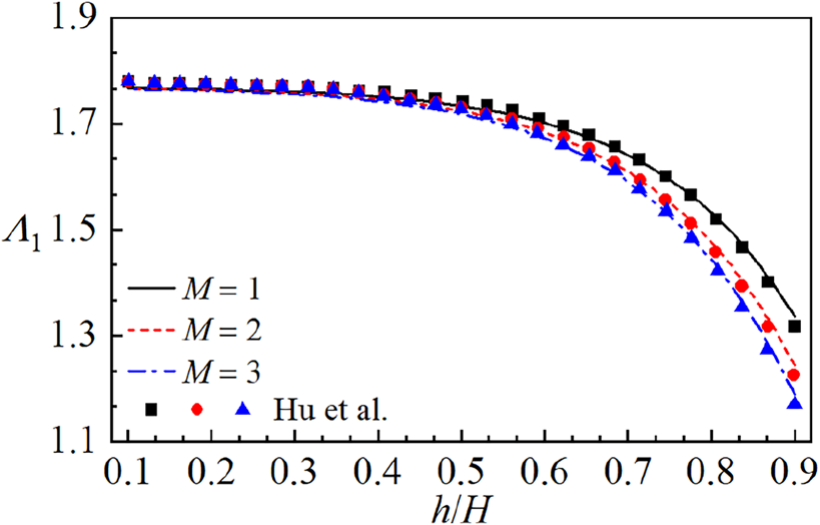
Comparison of the dimensionless first order sloshing frequency $\Lambda_{1}$ between present solutions and existing numerical solutions by Hu et al.^20^ with baffle numbers *M* = 1, 2, 3.

The vertical baffles are all positioned as the following forms in this subsection. The baffle is positioned at the container bottom with $a_{1} = B$ for *M* = 1; the baffles are, respectively, positioned at the container bottom with $a_{1} = {{2B} \mathord{\left/ {\vphantom {{2B} 3}} \right. \kern-0pt} 3}$ and ${{a_{2} = 4B} \mathord{\left/ {\vphantom {{a_{2} = 4B} 3}} \right. \kern-0pt} 3}$ for *M* = 2; and the baffles are, respectively, positioned at the container bottom with $a_{1} = {B \mathord{\left/ {\vphantom {B 2}} \right. \kern-0pt} 2}$, $a_{2} = B$ and ${{a_{3} = 3B} \mathord{\left/ {\vphantom {{a_{3} = 3B} 2}} \right. \kern-0pt} 2}$ for *M* = 3. The first three order sloshing frequency parameters $\Lambda_{n}^{2} \;(n = 1,\;2,\;3)$ versus the truncation item *N* under $M = 1,\;2,\;3$ are given in Table 4 to verify the convergence. The container parameters are listed as follows: $2B = 1\;{\text{m,}}$ $H = 1\;{\text{m}}$ and ${h \mathord{\left/ {\vphantom {h H}} \right. \kern-0pt} H} = 0.9.$ It is seen from Table 4 that the sloshing frequency converges rapidly with increase in truncated items *N*. The four significant digits could be guaranteed for the sloshing frequency when $N \ge 25$. Thus, the number of the truncated item $N = 25$ is considered in the present paper. In addition, Fig. 5 depicts the present results of the dimensionless first order sloshing frequency $\Lambda_{1}$ for *M* = 1, 2, 3 compared with the boundary element results^20^ under different baffle heights ${h \mathord{\left/ {\vphantom {h H}} \right. \kern-0pt} H}$. It is clear that present results display good agreement with numerical results.

**Table 4:** Convergence of sloshing frequency parameters $\Lambda_{n}^{2} \;(n = 1,\;2,\;3)$ versus the truncation item *N* with ${h \mathord{\left/ {\vphantom {h H}} \right. \kern-0pt} H}$ = 0.9.

*M*	*n*	*N* = 4	*N* = 7	*N* = 10	*N* = 13	*N* = 16	*N* = 19	*N* = 22	*N* = 25	*N* = 28
1	1	1.753	1.765	1.771	1.776	1.779	1.781	1.782	1.784	1.784
	2	6.283	6.283	6.283	6.283	6.283	6.283	6.283	6.283	6.283
	3	9.202	9.171	9.163	9.161	9.159	9.159	9.159	9.159	9.159
2	1	1.515	1.534	1.539	1.544	1.546	1.548	1.549	1.550	1.550
	2	4.895	4.930	4.942	4.949	4.954	4.957	4.960	4.962	4.963
	3	9.425	9.425	9.425	9.425	9.425	9.425	9.425	9.425	9.425
3	1	1.380	1.400	1.404	1.409	1.410	1.412	1.413	1.414	1.414
	2	4.624	4.667	4.679	4.687	4.692	4.695	4.697	4.699	4.701
	3	8.336	8.394	8.407	8.416	8.420	8.424	8.426	8.428	8.430

**Figure 6:**
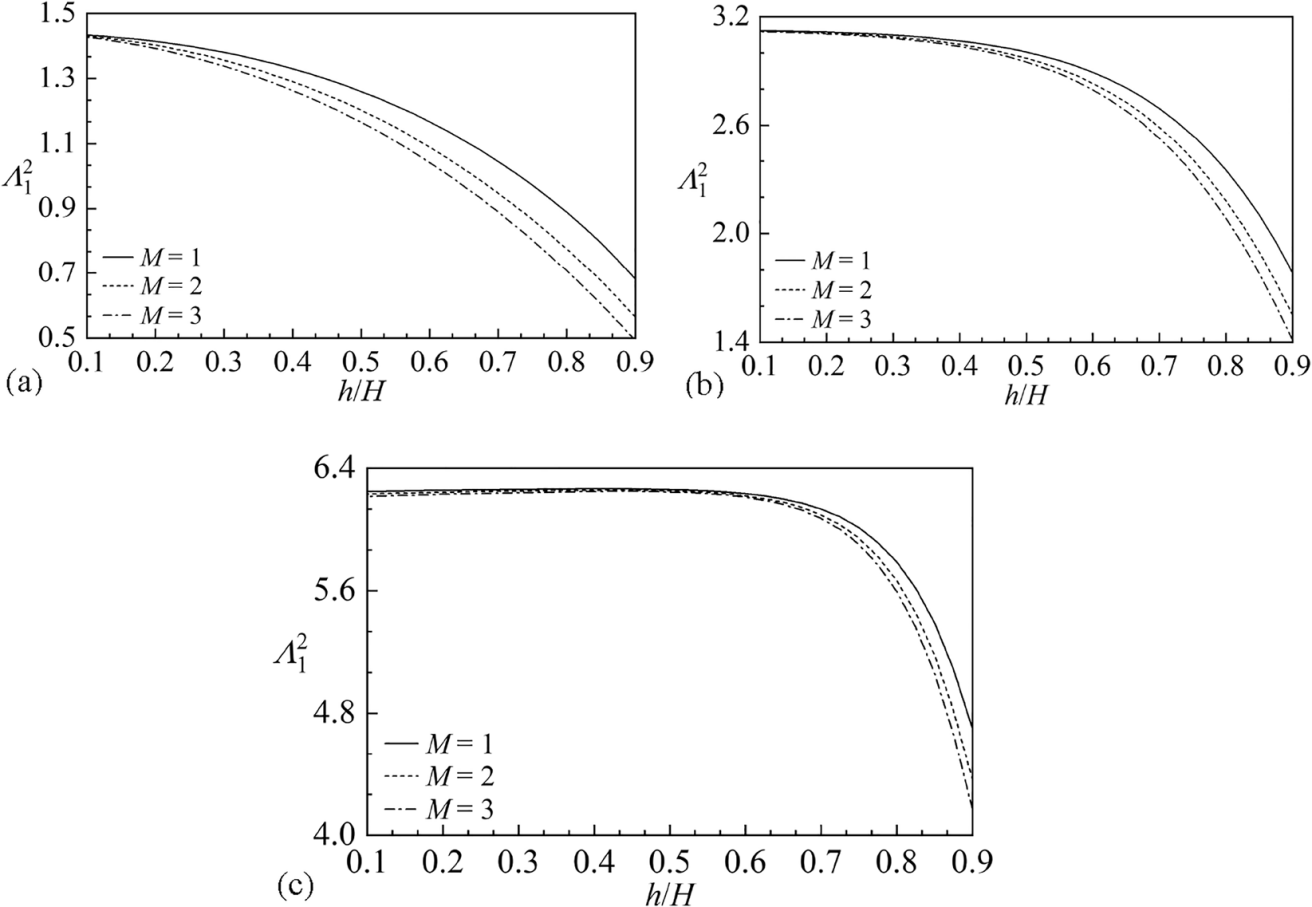
The first order sloshing frequency parameter $\Lambda_{\;1}^{\;2}$ versus the baffle height ${h \mathord{\left/ {\vphantom {h H}} \right. \kern-0pt} H}$ for various liquid heights ${H \mathord{\left/ {\vphantom {H B}} \right. \kern-0pt} B}$ and the baffle numbers *M*: (**a**) ${H \mathord{\left/ {\vphantom {H B}} \right. \kern-0pt} B} = 0.5;$ (**b**) ${H \mathord{\left/ {\vphantom {H B}} \right. \kern-0pt} B} = 1;$ (**c**) ${H \mathord{\left/ {\vphantom {H B}} \right. \kern-0pt} B} = 2.$

**Figure 7:**
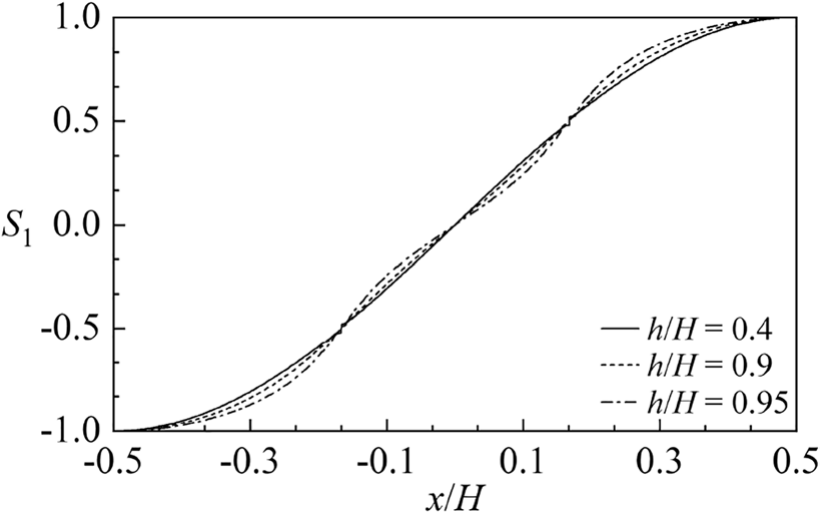
The first order sloshing mode shape $S_{1}$ corresponding to different baffle heights ${h \mathord{\left/ {\vphantom {h H}} \right. \kern-0pt} H}$ for *M* = 2.

**Figure 8:**
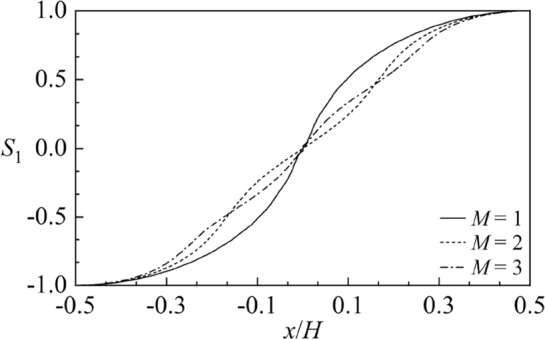
The first order sloshing mode shape $S_{1}$ corresponding to different baffle numbers *M* for ${h \mathord{\left/ {\vphantom {h H}} \right. \kern-0pt} H}$ = 0.95.

Figure 6 shows the first order sloshing frequency parameter $\Lambda_{1}^{2}$ versus the baffle height ${h \mathord{\left/ {\vphantom {h H}} \right. \kern-0pt} H}$ for different liquid heights ${H \mathord{\left/ {\vphantom {H B}} \right. \kern-0pt} B} = 0.5,\;1,\;2$ and baffle numbers *M* = 1, 2, 3 with $2B = 1\;{\text{m}}$. It is clear that as baffles gradually approach the free liquid surface, the sloshing frequency decreases significantly, replying that the existence of baffles close to free surface can effectively shift sloshing natural frequencies. The smaller the liquid height is, the more rapidly the discrepancy of the frequency results under various baffle numbers occurs. Besides, the increasing baffle number can induce the smaller sloshing frequency. Figure 7 gives the first order sloshing mode shape $S_{1}$ corresponding to the nondimensional baffle heights ${h \mathord{\left/ {\vphantom {h H}} \right. \kern-0pt} H}$ = 0.4, 0.9, 0.95 for $M = 2.$ It is clear that the discrepancy of sloshing mode shapes appears when the vertical baffles approach the free surface. Figure 8 shows the first order sloshing mode shape $S_{1}$ corresponding to different baffle numbers *M* = 1, 2, 3 for ${h \mathord{\left/ {\vphantom {h H}} \right. \kern-0pt} H}$ = 0.95. It can be observed from Figs. 7 and 8 that the baffle number exerts more significant impact on the sloshing mode compared with the baffle height.

### Response to horizontal harmonic excitation

**Figure 9:**
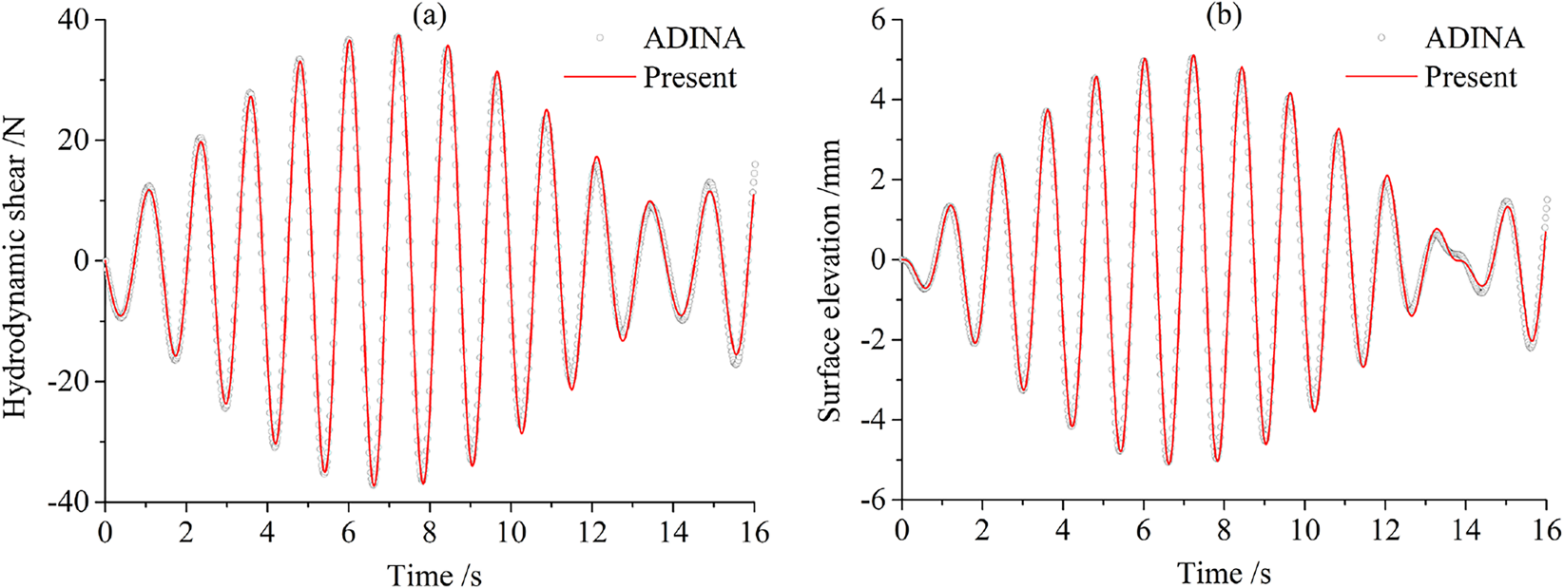
Comparison of responses of the container-liquid-baffle system: (**a**) the hydrodynamic shear force $F_{x}$; (**b**) the sloshing surface elevation $\eta$ at the left wall.

The numerical simulation of dynamic responses is conducted by using software ADINA to verify the proposed analytical method. The considered container parameters are given as follows: $M = 1,$ ${{a_{1} } \mathord{\left/ {\vphantom {{a_{1} } B}} \right. \kern-0pt} B} = 0.6,$ ${h \mathord{\left/ {\vphantom {h H}} \right. \kern-0pt} H} = 0.5,$ $2B = 1\;{\text{m}}$ and $H = 1\;{\text{m}}{.}$ The storage container is undergoing acceleration excitation $\ddot{u}(t) = - 0.01\sin \varpi t$ with the excitation frequency $\varpi = 5\;{\text{rad/s}}{.}$ In the ADINA model, the container and the baffle are simulated by four nodes 2-D solid elements; the liquid is simulated by four nodes 2-D fluid elements. A potential-based interface is applied to model boundary condition for free surface. The container-liquid-baffle model is found by 2525 potential-based liquid elements and 914 solid elements. The time history of the hydrodynamic shear $F_{x}$ and surface sloshing elevation $\eta$ at the left wall are plotted in Fig. 9. It is clear that present solutions display good agreement with finite element solutions.

**Figure 10:**
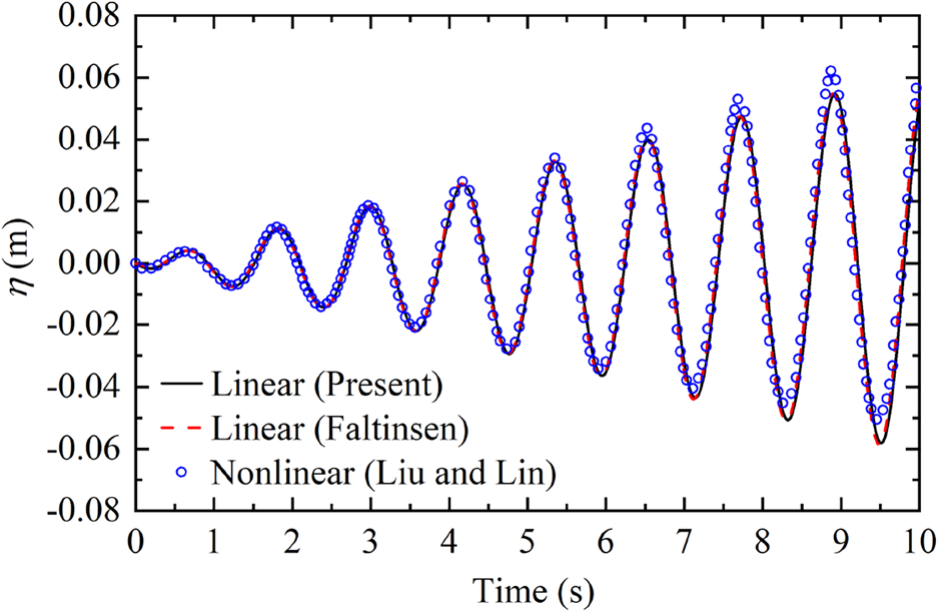
The present sloshing height $\eta$ at the right wall of the container under horizontal sinusoidal excitation in comparison with the reported linear and nonlinear results^33,34^.

To further validate the feasibility and correctness of the present method, the sloshing heights at the wall are compared with the linear and nonlinear solutions. Faltinsen^33^ presented the linear results of the sloshing surface elevation according to the potential flow theory; Liu and Lin^34^ constructed a numerical model to obtain the nonlinear surface elevation of a 2-D rectangular container without baffle. The container parameters are considered as $2B = 1\;{\text{m}}$, $H = 0.5\;{\text{m}}$ and ${{a_{1} } \mathord{\left/ {\vphantom {{a_{1} } B}} \right. \kern-0pt} B} = 0.5.$ The horizontal ground excitation $u(t) = X_{0} \sin \varpi t$ is utilized with $X_{0} = 0.002\;{\text{m}}$ and $\varpi = 5.29\;{\text{rad/s}}{.}$ Fig. 10 gives the present sloshing height $\eta$ at the right wall in comparison with linear and nonlinear results. It is clear from Fig. 10 that the present sloshing height is in conformity with the reported linear solution^33^. The relative error of the maximum sloshing height at the wall between present solutions and linear ones is −0.26%. The present solutions and reported nonlinear solutions^34^ also show good agreement in the first 4 s. As the excitation time continues to increase, the discrepancy between present and nonlinear solutions gradually occurs. The wave peak amplitude is greater than the wave trough amplitude since the nonlinear sloshing effect is considered by Liu and Lin^34^. Besides, the present first-order frequency of convective sloshing is 5.316 rad/s. The relative difference of the maximum sloshing height at the wall between present solutions and nonlinear ones is −9.07%. This comparison implies that under the circumstance of the small amplitude of tank motion, the present results are still close to nonlinear results even when the discrepancy between the excitation frequency and first-order convective sloshing frequency is reduced to 0.49%.

**Figure 11:**
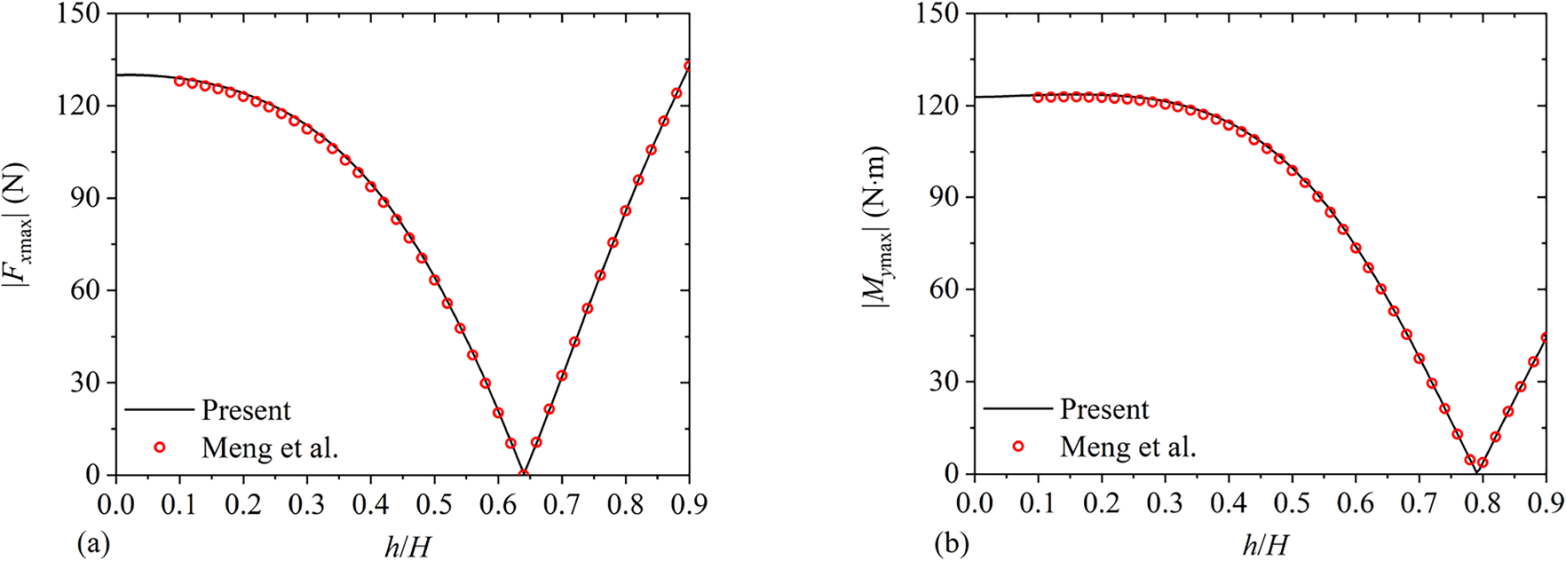
The present hydrodynamic responses of the container under horizontal sinusoidal excitation in comparison with the exact results^29^: (**a**) the amplitude of the hydrodynamic shear force |*F*_*x*max_|; (**b**) the amplitude of the hydrodynamic overturning moment |*M*_*y*max_|.

Furthermore, Meng et al.^29^ utilized a semi-analytical approach to study hydrodynamic responses of a rectangular container with a vertical baffle. The container parameters are considered as $2B = 1\;{\text{m}}$, $H = 1.0\;{\text{m}}$ and ${{a_{1} } \mathord{\left/ {\vphantom {{a_{1} } B}} \right. \kern-0pt} B} = 1.$ The horizontal sinusoidal excitation is utilized with $X_{0} = 0.005\;{\text{m}}$ and $\varpi = 6\;{\text{rad/s}}{.}$ Fig. 11 depicts amplitudes of the hydrodynamic shear force |*F*_*x*max_| and hydrodynamic overturning moment |*M*_*y*max_| versus the baffle height ${h \mathord{\left/ {\vphantom {h H}} \right. \kern-0pt} H}$ in comparison with the reported exact results^29^. It is clear that the present results are in good accordance with available results.

**Figure 12:**
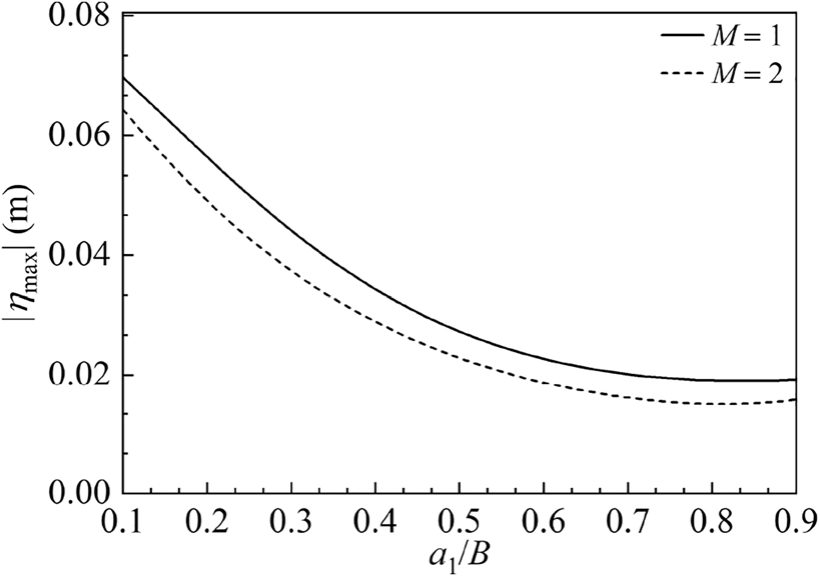
The amplitude variations of the sloshing height at the right wall |*η*_max_| versus the baffle position $a_{1} /B$ for the baffle number *M* = 1, 2.

**Figure 13:**
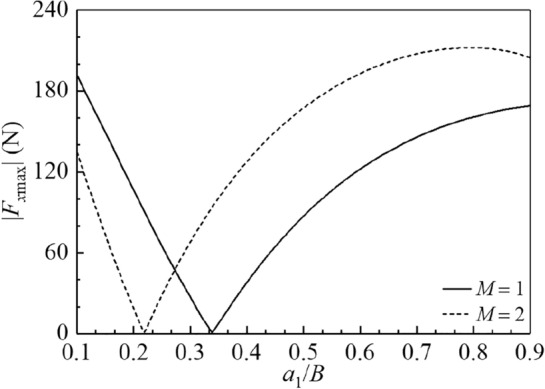
The amplitude variations of the hydrodynamic shear |*F*_*x*max_| versus the baffle position $a_{1} /B$ for the baffle number *M* = 1, 2.

**Figure 14:**
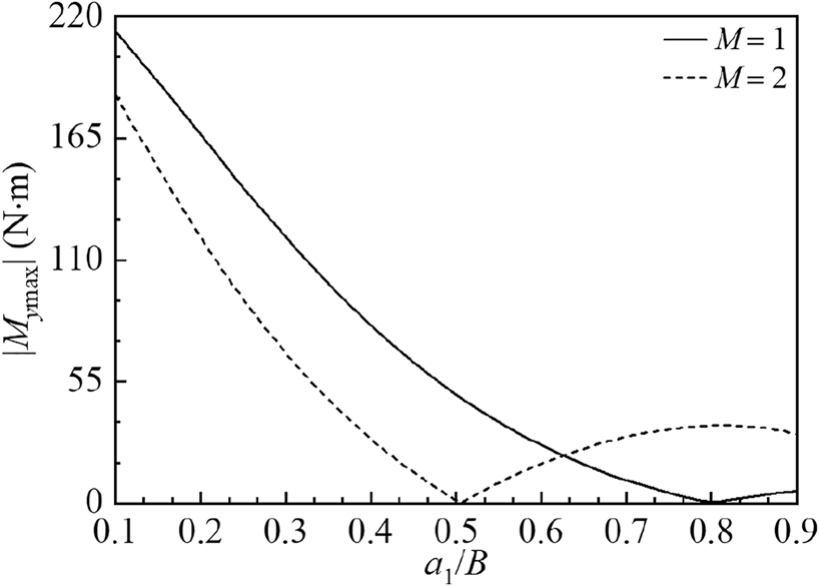
The amplitude variations of the hydrodynamic overturning moment |*M*_*y*max_| versus the baffle position $a_{1} /B$ for the baffle number *M* = 1, 2.

The effects of baffle positions $${{a_{1} } \mathord{\left/ {\vphantom {{a_{1} } B}} \right. \kern-0pt} B}$$ on amplitudes of the sloshing height at the right wall |*η*_max_|, hydrodynamic shear |*F*_*x*max_| and hydrodynamic moment |*M*_*y*max_| are depicted in Figs. 12, 13 and 14 for $$M = 1,\;2,$$$$2B = 1\;{\text{m,}}$$ $$H = 1\;{\text{m,}}$$ $$X_{0} = 0.01\;{\text{m}}$$ and $$\overline{\omega } = 6\;{\text{rad/s}}.$$ The baffles are located symmetrically regarding the container bottom center for $$M = 2$$ and $${h \mathord{\left/ {\vphantom {h H}} \right. \kern-0pt} H} = 0.8.$$ Figure 12 displays curves of amplitude of the sloshing height at the right wall |*η*_max_| for $${{a_{1} } \mathord{\left/ {\vphantom {{a_{1} } B}} \right. \kern-0pt} B}$$. It is seen that as the baffle moves horizontally from the vicinity of the left wall to the bottom center of the container, |*η*_max_| decreases significantly. Besides, |*η*_max_| further declines by increasing the vertical baffle number. Figure 13 shows the amplitude variations of |*F*_*x*max_| versus $${{a_{1} } \mathord{\left/ {\vphantom {{a_{1} } B}} \right. \kern-0pt} B}$$. For $$M = 1,$$ as the bottom-mounted baffle moves horizontally from the vicinity of the left wall to the bottom center of the container, |*F*_*x*max_| first decreases and then increases, reaching zero at $${{a_{1} } \mathord{\left/ {\vphantom {{a_{1} } B}} \right. \kern-0pt} B}$$ = 0.338; for $$M = 2,$$ the bottom-mounted vertical baffles exert great impact on |*F*_*x*max_|. The zero point position of |*F*_*x*max_| for $$M = 2$$ moves towards the left container wall compared with the zero point position of |*F*_*x*max_| for $$M = 1.$$ Figure 14 shows the amplitude variations of |*M*_*y*max_| versus the baffle position $${{a_{1} } \mathord{\left/ {\vphantom {{a_{1} } B}} \right. \kern-0pt} B}$$. It is seen that with the increase of $${{a_{1} } \mathord{\left/ {\vphantom {{a_{1} } B}} \right. \kern-0pt} B}$$, |*M*_*y*max_| first declines significantly and then slowly increases. The values of |*M*_*y*max_| reach zero, respectively, at $${{a_{1} } \mathord{\left/ {\vphantom {{a_{1} } B}} \right. \kern-0pt} B}$$ = 0.802 and $${{a_{1} } \mathord{\left/ {\vphantom {{a_{1} } B}} \right. \kern-0pt} B}$$ = 0.506 for $$M = 1$$ and $$M = 2.$$

**Figure 15:**
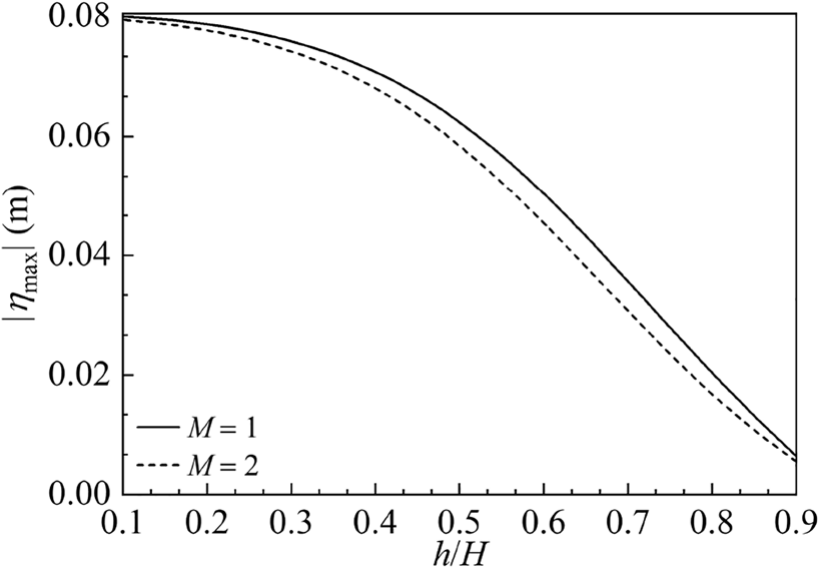
The amplitude variations of the sloshing height at the right wall |*η*_max_| versus the baffle height $h/H$ for the baffle number *M* = 1, 2.

**Figure 16:**
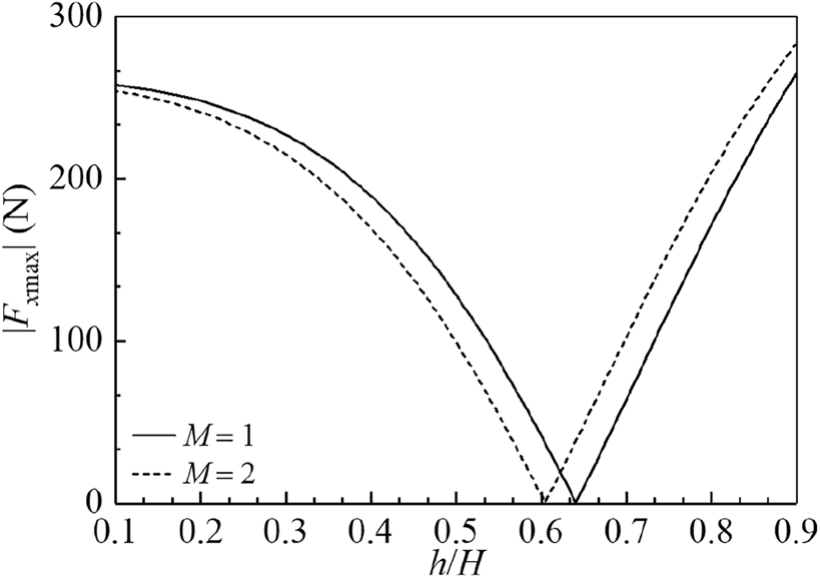
The amplitude variations of the hydrodynamic shear |*F*_*x*max_| versus the baffle height $h/H$ for the baffle number *M* = 1, 2.

**Figure 17:**
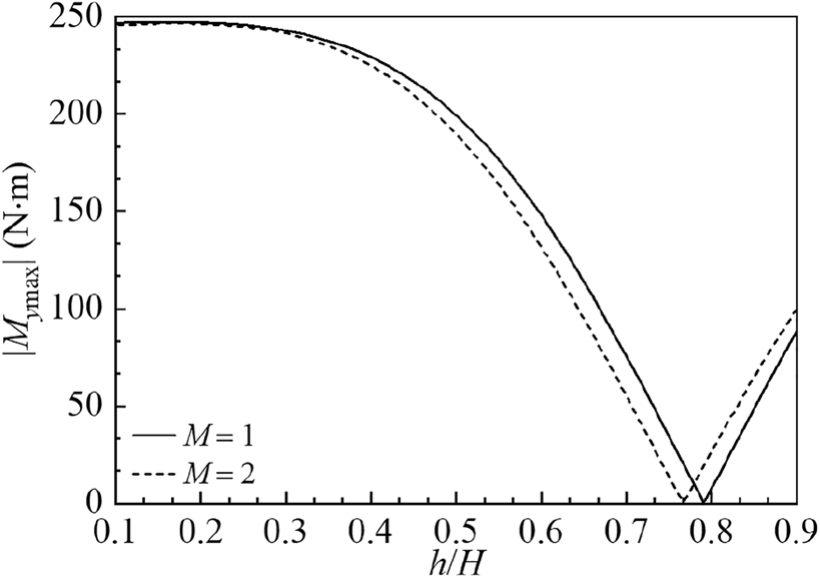
The amplitude variations of the hydrodynamic overturning moment |*M*_*y*max_| versus the baffle height $h/H$ for the baffle number* M* = 1, 2.

The amplitude variations of the sloshing height at the right wall |*η*_max_|, hydrodynamic shear |*F*_*x*max_| and hydrodynamic moment |*M*_*y*max_| versus the baffle height $${h \mathord{\left/ {\vphantom {h H}} \right. \kern-0pt} H}$$ are illustrated in Figs. 15, 16 and 17 for $$M = 1,\;2,$$$$2B = 1\;{\text{m}}$$ and $$H = 1\;{\text{m}}{.}$$ The utilized horizontal harmonic excitation is considered as $$X_{0} = 0.01\;{\text{m}}$$ and $$\overline{\omega } = 6\;{\text{rad/s}}.$$ The vertical baffles are sequentially located at the $$M + 1$$ equal-dividing points at the container bottom with the number of baffles *M*. It is found in Fig. 15 that the baffle height exerts great impact on |*η*_max_|. With increase in the baffle height, |*η*_max_| monotonically decreases for $$M = 1,\;2$$. It is clear in Figs. 16 and 17 that by increasing the baffle height, |*F*_*x*max_| and |*M*_*y*max_| first decrease and then increase. The values of |*F*_*x*max_| reach zero, respectively, at $${h \mathord{\left/ {\vphantom {h H}} \right. \kern-0pt} H} = 0.641$$ and $${h \mathord{\left/ {\vphantom {h H}} \right. \kern-0pt} H} = 0.605$$ for $$M = 1$$ and $$M = 2$$; and the values of |*M*_*y*max_| reach zero, respectively, at $${h \mathord{\left/ {\vphantom {h H}} \right. \kern-0pt} H} = 0.791$$ and $${h \mathord{\left/ {\vphantom {h H}} \right. \kern-0pt} H} = 0.767$$ for $$M = 1$$ and $$M = 2.$$

### Response to horizontal seismic excitation

**Figure 18:**
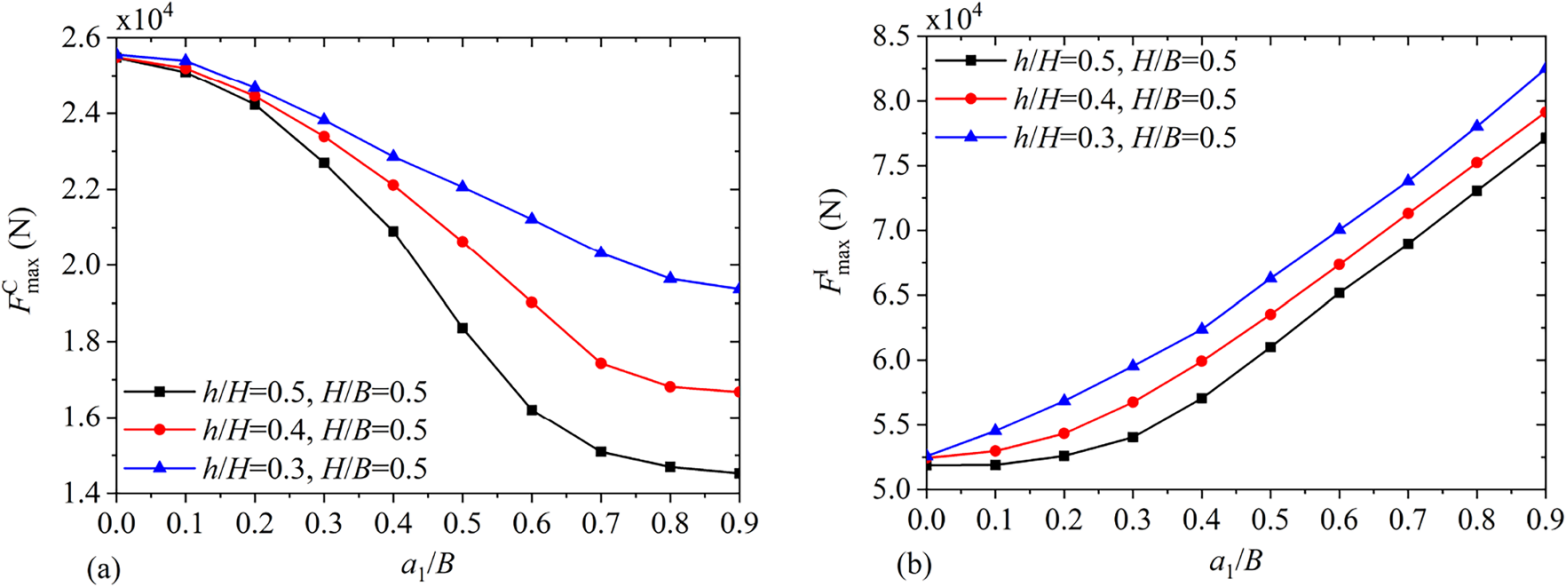
The maximum values of hydrodynamic responses versus the vertical baffle position ${{a_{1} } \mathord{\left/ {\vphantom {{a_{1} } B}} \right. \kern-0pt} B}$: (**a**) convective shear $F_{\max }^{{\text{C}}}$; (**b**) impulsive shear $F_{\max }^{{\text{I}}}$.

**Figure 19:**
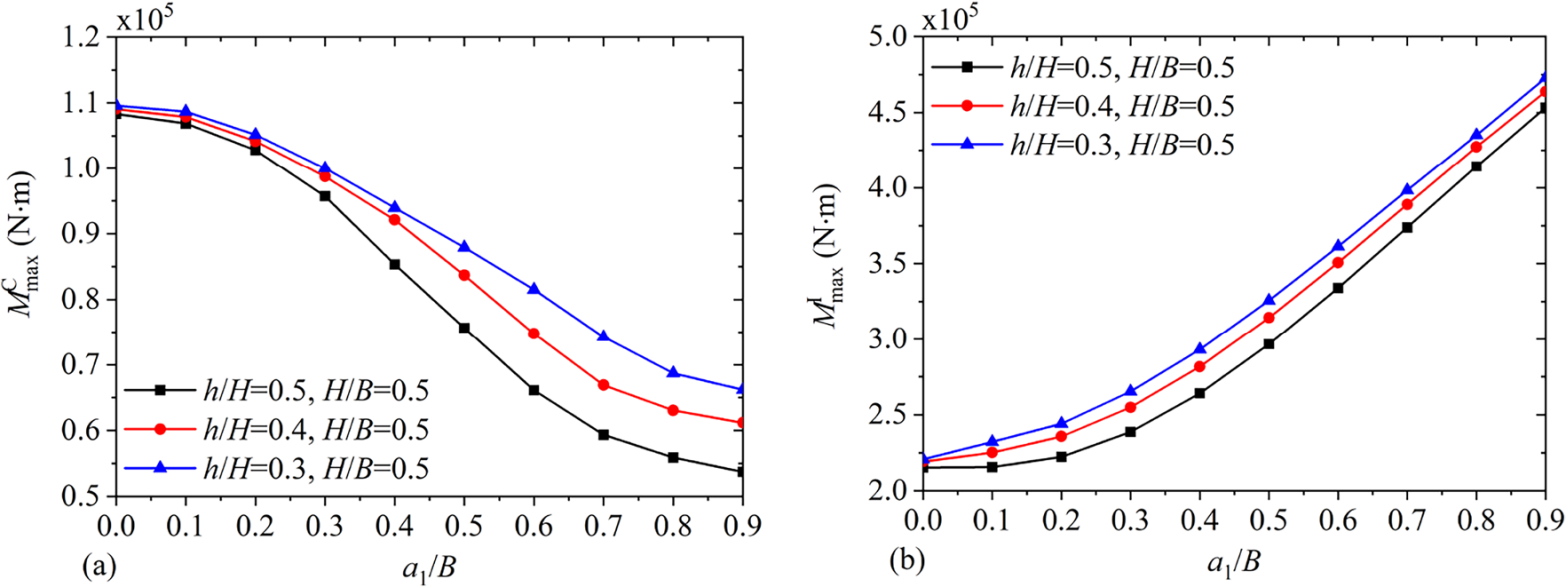
The maximum values of hydrodynamic responses versus the vertical baffle position ${{a_{1} } \mathord{\left/ {\vphantom {{a_{1} } B}} \right. \kern-0pt} B}$: (**a**) convective moment $M_{\max }^{{\text{C}}}$; (**b**) impulsive moment $M_{\max }^{{\text{I}}}$.

By utilizing the data, such as those given in Tables 2 and 3 as well as Fig. 4, the maximum values of the convective and impulsive components of hydrodynamic responses can be easily obtained for various baffle positions, baffle heights and liquid heights under earthquake excitations for $M = 1$ and $2B = 10\;{\text{m}}$. The utilized seismic record is the 30 s Kobe wave excitation recorded in 1995 at Kobe Japanese Meteorological Agency Station (KJM-000) with the time interval 0.02 s. The acceleration peak value is 0.834*g*. As shown in Fig. 18, the maximum values of the convective shear $F_{\max }^{{\text{C}}}$ and impulsive shear $F_{\max }^{{\text{I}}}$ vary versus the vertical baffle position ${{a_{1} } \mathord{\left/ {\vphantom {{a_{1} } B}} \right. \kern-0pt} B}$ for ${h \mathord{\left/ {\vphantom {h H}} \right. \kern-0pt} H} = 0.3,\;0.4,\;0.5$ and ${H \mathord{\left/ {\vphantom {H B}} \right. \kern-0pt} B} = 0.5$. It is clear that as the baffle moves towards the bottom center, $F_{\max }^{{\text{C}}}$ declines monotonically, however, $F_{\max }^{{\text{I}}}$ increases. Figure 19 shows the maximum values of the convective moment $M_{\max }^{{\text{C}}}$ and impulsive moment $M_{\max }^{{\text{I}}}$ versus ${{a_{1} } \mathord{\left/ {\vphantom {{a_{1} } B}} \right. \kern-0pt} B}$ for ${h \mathord{\left/ {\vphantom {h H}} \right. \kern-0pt} H} = 0.3,\;0.4,\;0.5$ and ${H \mathord{\left/ {\vphantom {H B}} \right. \kern-0pt} B} = 0.5$. It is seen that with the increase of ${{a_{1} } \mathord{\left/ {\vphantom {{a_{1} } B}} \right. \kern-0pt} B}$, $M_{\max }^{{\text{C}}}$ decreases monotonically, however, $M_{\max }^{{\text{I}}}$ increases.

**Figure 20:**
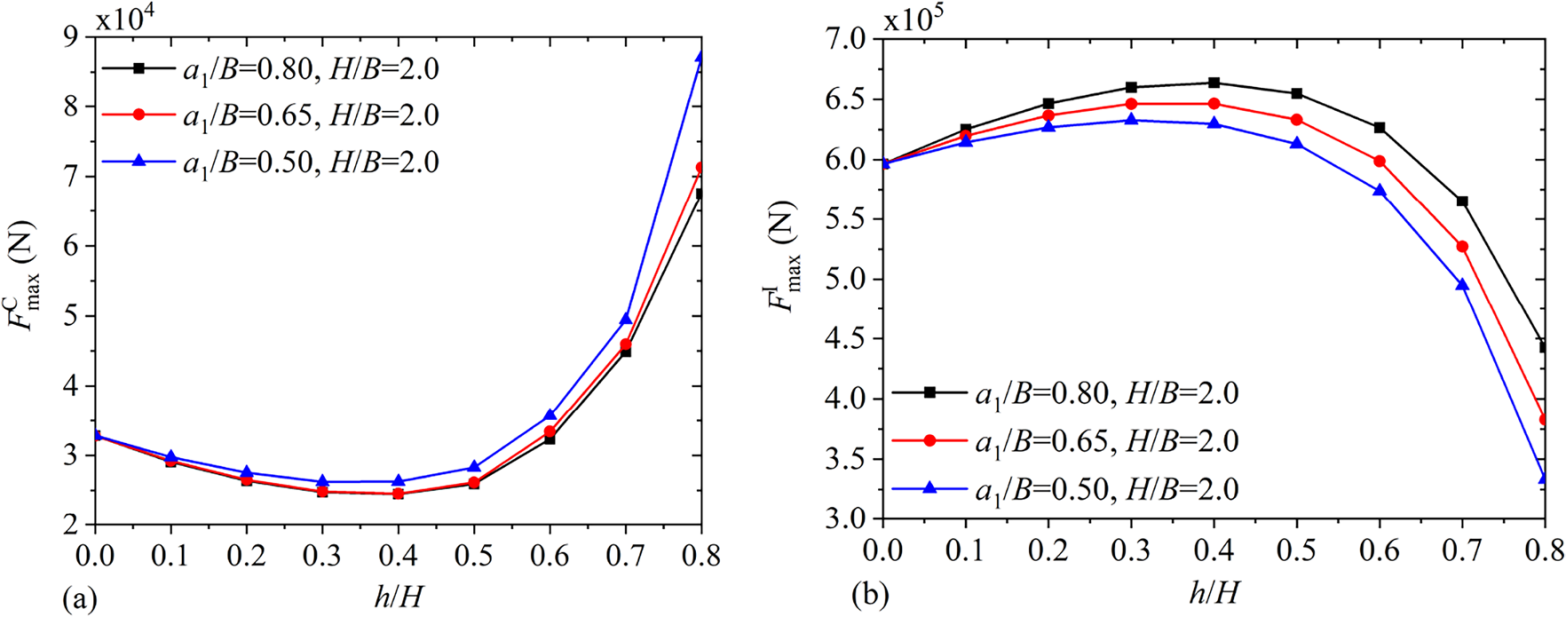
The maximum values of hydrodynamic responses versus the vertical baffle height ${h \mathord{\left/ {\vphantom {h H}} \right. \kern-0pt} H}$: (**a**) convective shear $F_{\max }^{{\text{C}}}$; (**b**) impulsive shear $F_{\max }^{{\text{I}}}$.

**Figure 21:**
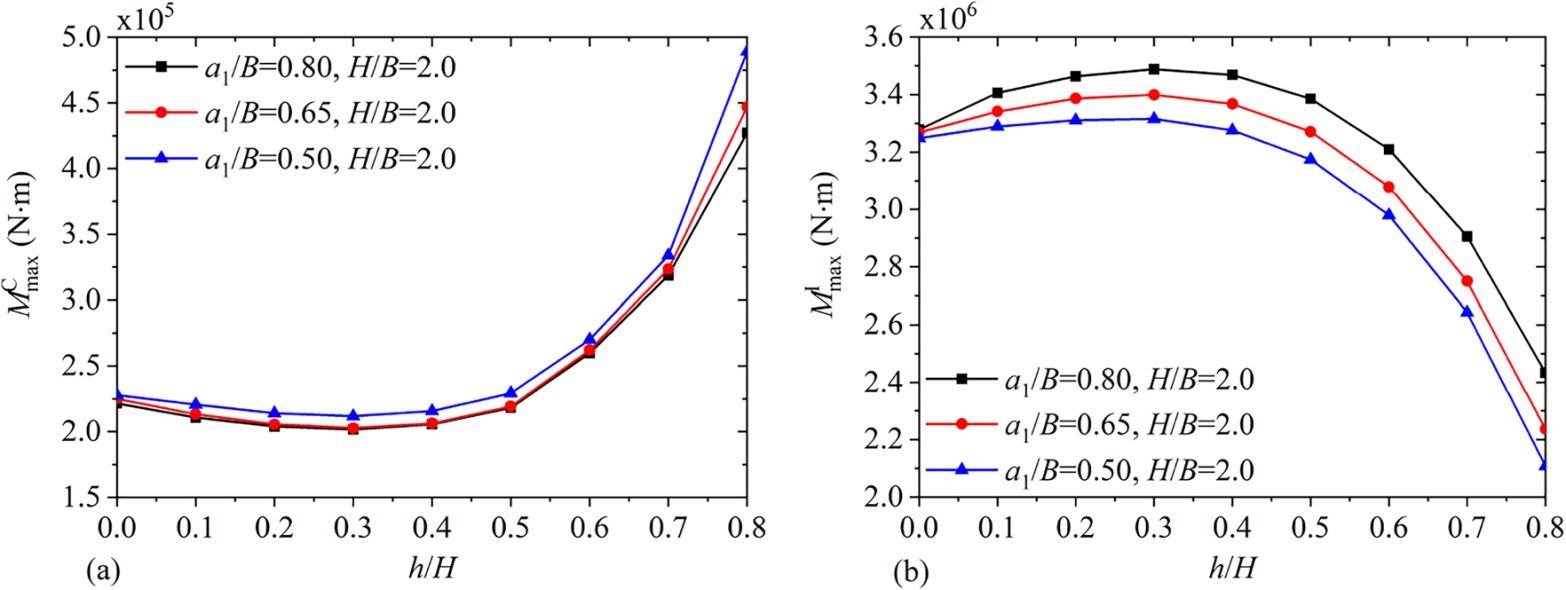
The maximum values of hydrodynamic responses versus the vertical baffle height ${h \mathord{\left/ {\vphantom {h H}} \right. \kern-0pt} H}$: (**a**) convective moment $M_{\max }^{{\text{C}}}$; (**b**) impulsive moment $M_{\max }^{{\text{I}}}$.

**Figure 22:**
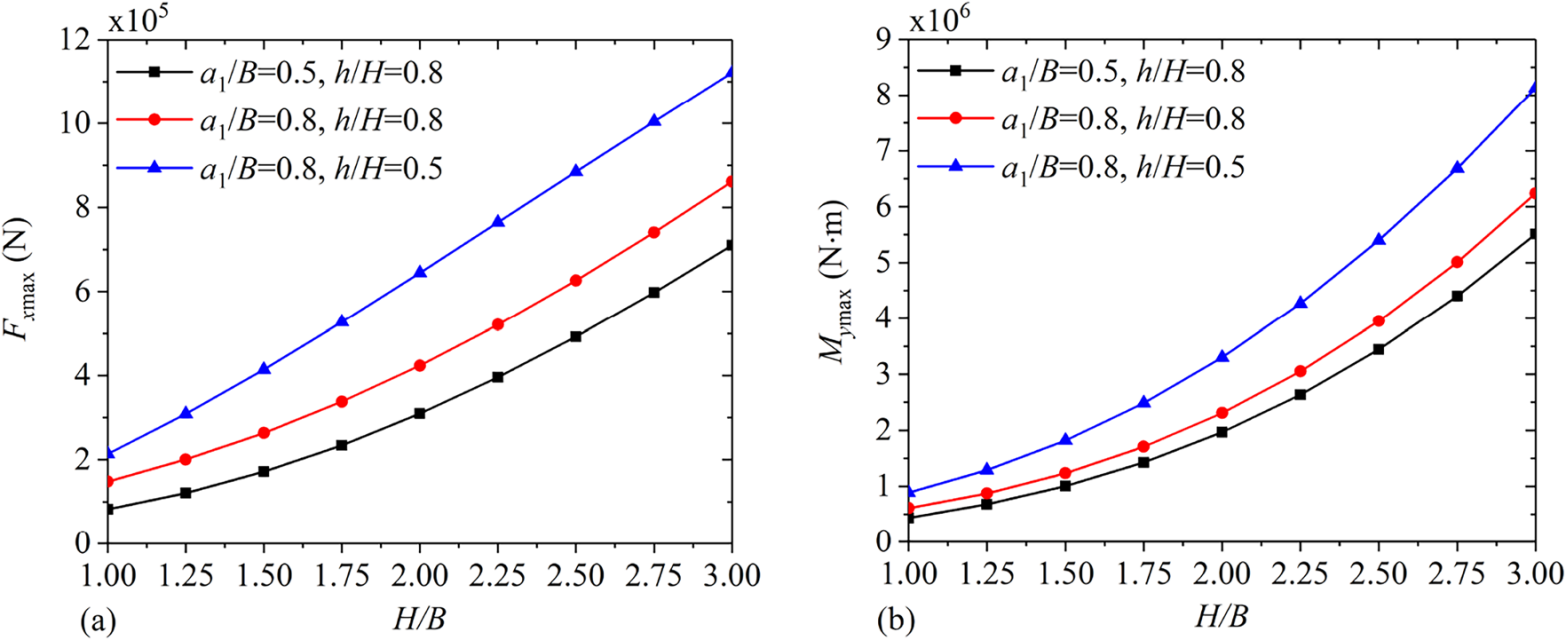
The maximum values of hydrodynamic responses versus the liquid height ${H \mathord{\left/ {\vphantom {H B}} \right. \kern-0pt} B}$: (**a**) the shear force $F_{x\max }$; (**b**) the overturning moment $M_{y\max }$.

Figures 20 and 21 depict the variations of $F_{\max }^{{\text{C}}}$, $F_{\max }^{{\text{I}}}$, $M_{\max }^{{\text{C}}}$ and $M_{\max }^{{\text{I}}}$ under different baffle heights ${h \mathord{\left/ {\vphantom {h H}} \right. \kern-0pt} H}$ for ${{a_{1} } \mathord{\left/ {\vphantom {{a_{1} } B}} \right. \kern-0pt} B} = 0.5,\;0.65,\;0.8$ and ${H \mathord{\left/ {\vphantom {H B}} \right. \kern-0pt} B} = 2.0$. It is clear that through increasing the vertical baffle height, the maximum convective and impulsive components of hydrodynamic responses show the non-monotonical variation. Figure 22 illustrates maximum values of the hydrodynamic shear $F_{x\max }$ and moment $M_{y\max }$ with different liquid heights ${H \mathord{\left/ {\vphantom {H B}} \right. \kern-0pt} B}$ for ${{a_{1} } \mathord{\left/ {\vphantom {{a_{1} } B}} \right. \kern-0pt} B} = 0.5,\;0.8$ and ${h \mathord{\left/ {\vphantom {h H}} \right. \kern-0pt} H} = 0.5,\;0.8$. It is clear that with increase in the liquid height, $F_{x\max }$ and $M_{y\max }$ both increase monotonically.

## Conclusions

An analytical equivalent model of the continuous liquid sloshing in a 2-D rectangular container equipped with rigid vertical baffles undergoing horizontal excitation is proposed. The sloshing properties are solved by utilizing the subdomain partition approach. The dynamic responses are calculated on the basis of the mode superposition approach. By producing the same hydrodynamic shear and moment obtained from the proposed model as those of the original container system, detailed expressions of the convective and impulsive masses as well as corresponding positions are given. The variation laws of model parameters are discussed regarding normalized baffle positions, baffle heights and liquid heights. Through utilizing the proposed model, the baffle effect on convective and impulsive responses could be analyzed, which provides the better comprehending of sloshing mechanism in a storage container. The critical findings can be concluded as follows:

1. The existence of baffles approaching free surface can effectively reduce sloshing frequencies. The baffle number exerts effect on sloshing properties and responses. The sloshing height amplitude declines remarkably with increase in the baffle height.
2. As the baffle moves horizontally towards the bottom center and/or approaches liquid free surface, amplitudes of the hydrodynamic shear and moment first decrease, reaching zero values and then increase, showing the non-monotonic variations.
3. As the baffle moves horizontally from the vicinity of the wall to the bottom center of the container, maxima of the convective shear and moment both decline, however, impulsive components increase monotonically.
4. By increasing the vertical baffle height, maximum convective and impulsive components of hydrodynamic responses show the non-monotonical variations.

## Data Availability

The datasets used and/or analysed during the current study available from the corresponding author on reasonable request.
